# Obesity, metabolic factors and risk of different histological types of lung cancer: A Mendelian randomization study

**DOI:** 10.1371/journal.pone.0177875

**Published:** 2017-06-08

**Authors:** Robert Carreras-Torres, Mattias Johansson, Philip C. Haycock, Kaitlin H. Wade, Caroline L. Relton, Richard M. Martin, George Davey Smith, Demetrius Albanes, Melinda C. Aldrich, Angeline Andrew, Susanne M. Arnold, Heike Bickeböller, Stig E. Bojesen, Hans Brunnström, Jonas Manjer, Irene Brüske, Neil E. Caporaso, Chu Chen, David C. Christiani, W. Jay Christian, Jennifer A. Doherty, Eric J. Duell, John K. Field, Michael P. A. Davies, Michael W. Marcus, Gary E. Goodman, Kjell Grankvist, Aage Haugen, Yun-Chul Hong, Lambertus A. Kiemeney, Erik H. F. M. van der Heijden, Peter Kraft, Mikael B. Johansson, Stephen Lam, Maria Teresa Landi, Philip Lazarus, Loïc Le Marchand, Geoffrey Liu, Olle Melander, Sungshim L. Park, Gad Rennert, Angela Risch, Eric B. Haura, Ghislaine Scelo, David Zaridze, Anush Mukeriya, Milan Savić, Jolanta Lissowska, Beata Swiatkowska, Vladimir Janout, Ivana Holcatova, Dana Mates, Matthew B. Schabath, Hongbing Shen, Adonina Tardon, M Dawn Teare, Penella Woll, Ming-Sound Tsao, Xifeng Wu, Jian-Min Yuan, Rayjean J. Hung, Christopher I. Amos, James McKay, Paul Brennan

**Affiliations:** 1 Section of Genetics, International Agency for Research on Cancer (IARC), Lyon, France; 2 MRC Integrative Epidemiology Unit, School of Social and Community Medicine, University of Bristol, Bristol, United Kingdom; 3 National Institute for Health Research Biomedical Research Unit in Nutrition, Diet and Lifestyle at University Hospitals Bristol NHS Foundation Trust and the University of Bristol, Bristol, United Kingdom; 4 Metabolic Epidemiology Branch, DCEG, National Cancer Institute, NIH, Rockville, Maryland, United States of America; 5 Department of Thoracic Surgery, Division of Epidemiology, Vanderbilt University Medical Center, Nashville, Tennessee, United States of America; 6 Norris Cotton Cancer Center, Lebanon, New Hampshire, United States of America; 7 University of Kentucky Markey Cancer Center, Lexington, Kentucky, United States of America; 8 University Medical Center Göettingen, Göttingen, Germany; 9 Faculty of Health and Medical Sciences, University of Copenhagen, Copenhagen, Denmark; 10 Laboratory Medicine Region Skåne, Department of Clinical Sciences Lund, Lund University, Lund, Sweden; 11 Helmholtz Zentrum München, Munich, Germany; 12 Genetic Epidemiology Branch, DCEG, National Cancer Institute, NIH, Rockville, Maryland, United States of America; 13 Program in Epidemiology, Division of Public Health Sciences, Fred Hutchinson Cancer Research Center, Seattle, Washington, United States of America; 14 Departments of Environmental Health and Epidemiology, Harvard T. H. Chan School of Public Health and Department of Medicine, Massachusetts General Hospital/Harvard Medical School, Boston, Massachusetts, United States of America; 15 Department of Epidemiology, College of Public Health, University of Kentucky, Lexington, Kentucky, United States of America; 16 Department of Epidemiology, Geisel School of Medicine, Dartmouth College, Lebanon, New Hampshire, United States of America; 17 Unit of Nutrition and Cancer, Cancer Epidemiology Research Program, Catalan Institute of Oncology (ICO-IDIBELL), Barcelona, Spain; 18 Roy Castle Lung Cancer Research Programme, Department of Molecular and Clinical Cancer Medicine, Institute of Translational Medicine, The University of Liverpool, Liverpool, United Kingdom; 19 Fred Hutchinson Cancer Research Center, Seattle, Washington, United States of America; 20 Department of Medical Biosciences, Umeå University, Umeå, Sweden; 21 National Institute of Occupational Health, Oslo, Norway; 22 Department of Preventive Medicine, Seoul National University College of Medicine, Seoul, Korea; 23 Radboud University Medical Center, Nijmegen, The Nederlands; 24 Department of Epidemiology, Harvard T.H. Chan School of Public Health, Boston, Massachusetts, United States of America; 25 British Columbia Cancer Agency, Vancouver, British Columbia, Canada; 26 Washington State University College of Pharmacy, Spokane, Washington, United States of America; 27 Epidemiology Program, University of Hawaii Cancer Center, Honolulu, Hawaii, United States of America; 28 Ontario Cancer Institute, Princess Margaret Cancer Center, Toronto, Ontario, Canada; 29 Department of Clinical Sciences Malmö, Lund University, Lund, Sweden; 30 Department of Internal Medicine, Skåne University Hospital, Malmö, Sweden; 31 Norris Comprehensive Cancer Center, Keck School of Medicine, University of Southern California, Los Angeles, California, United States of America; 32 Clalit National Cancer Control Center and Department of Community Medicine and Epidemiology, Carmel Medical Center and B. Rappaport Faculty of Medicine, Technion-Israel Institute of Technology, Haifa, Israel; 33 University of Salzburg and Cancer Cluster Salzburg, Salzburg, Austria; 34 Department of Thoracic Oncology, H. Lee Moffitt Cancer Center and Research Institute, Tampa, Florida, United States of America; 35 Russian N.N. Blokhin Cancer Research Centre, Moscow, The Russian Federation; 36 Department of Thoracic Surgery, Clinical Center of Serbia, Belgrade, Serbia; 37 Department of Cancer Epidemiology and Prevention, Maria Sklodowska-Curie Institute – Oncology Center, Warsaw, Poland; 38 Nofer Institute of Occupational Medicine, Department of Environmental Epidemiology, Lodz, Poland; 39 Department of Epidemiology and Public Health, Faculty of Medicine, University of Ostrava, Ostrava, Czech Republic; 40 Institute of Public Health and Preventive Medicine, Charles University, 2nd Faculty of Medicine, Prague, Czech Republic; 41 National Institute of Public Health, Bucharest, Romania; 42 Department of Cancer Epidemiology, H. Lee Moffitt Cancer Center and Research Institute, Tampa, Florida, United States of America; 43 Department of Epidemiology and Biostatistics, Jiangsu Key Lab of Cancer Biomarkers, Prevention and Treatment, Collaborative Innovation Center for Cancer Personalized Medicine, School of Public Health, Nanjing Medical University, Nanjing, China; 44 Instituto Universitario de Oncología del Principado de Asturias (IUOPA), University of Oviedo and CIBERESP, Oviedo, Spain; 45 University of Sheffield, Sheffield, United Kingdom; 46 Princess Margaret Cancer Center, Toronto, Canada; 47 The University of Texas MD Anderson Cancer Center, Houston, Texas, United States of America; 48 Division of Cancer Control and Population Science, University of Pittsburgh Cancer Institute; and Department of Epidemiology, University of Pittsburgh Graduate School of Public Health, Pittsburgh, Pennsylvania, United States of America; 49 Lunenfeld-Tanenbaum Research Institute of Mount Sinai Hospital, Toronto, Canada; 50 Department of Biomedical Data Science, Geisel School of medicine, Dartmouth College, Lebanon, New Hampshire, United States of America; Shanghai Diabetes Institute, CHINA

## Abstract

**Background:**

Assessing the relationship between lung cancer and metabolic conditions is challenging because of the confounding effect of tobacco. Mendelian randomization (MR), or the use of genetic instrumental variables to assess causality, may help to identify the metabolic drivers of lung cancer.

**Methods and findings:**

We identified genetic instruments for potential metabolic risk factors and evaluated these in relation to risk using 29,266 lung cancer cases (including 11,273 adenocarcinomas, 7,426 squamous cell and 2,664 small cell cases) and 56,450 controls. The MR risk analysis suggested a causal effect of body mass index (BMI) on lung cancer risk for two of the three major histological subtypes, with evidence of a risk increase for squamous cell carcinoma (odds ratio (OR) [95% confidence interval (CI)] = 1.20 [1.01–1.43] and for small cell lung cancer (OR [95%CI] = 1.52 [1.15–2.00]) for each standard deviation (SD) increase in BMI [4.6 kg/m^2^]), but not for adenocarcinoma (OR [95%CI] = 0.93 [0.79–1.08]) (*P*_*heterogeneity*_ *= 4*.*3x10*^*-3*^). Additional analysis using a genetic instrument for BMI showed that each SD increase in BMI increased cigarette consumption by 1.27 cigarettes per day (P = 2.1x10^-3^), providing novel evidence that a genetic susceptibility to obesity influences smoking patterns. There was also evidence that low-density lipoprotein cholesterol was inversely associated with lung cancer overall risk (OR [95%CI] = 0.90 [0.84–0.97] per SD of 38 mg/dl), while fasting insulin was positively associated (OR [95%CI] = 1.63 [1.25–2.13] per SD of 44.4 pmol/l). Sensitivity analyses including a weighted-median approach and MR-Egger test did not detect other pleiotropic effects biasing the main results.

**Conclusions:**

Our results are consistent with a causal role of fasting insulin and low-density lipoprotein cholesterol in lung cancer etiology, as well as for BMI in squamous cell and small cell carcinoma. The latter relation may be mediated by a previously unrecognized effect of obesity on smoking behavior.

## Introduction

Lung cancer is the leading cause of cancer mortality [1]. Most lung cancers are caused by tobacco smoking [2], although associations have also been reported with a range of inflammatory and metabolic conditions. Observational studies have indicated an inverse relationship for both body mass index (BMI) [3–7] and lipid levels [8,9], as well as a positive correlation with dietary glycemic index [10] and insulin levels [11], with lung cancer risk. However, given the strong effect of tobacco smoking on lung cancer risk, and the well described association between tobacco consumption and alterations in body weight [12–14], traditional observational studies are unlikely to fully account for the confounding effect of tobacco exposure when describing the relationship between lung cancer and obesity or metabolic conditions.

Mendelian randomization (MR) is an analytical approach based on genetic markers of an exposure (i.e. an instrumental variable) that is less sensitive to reverse causation and confounding than traditional regression analyses in observational studies [15]. Inherited gene variants associated with the risk factors of interest should act as unconfounded markers of those risk factors, assuming an absence of pleiotropy [16]. In this instance, an association between the genetic variant and the outcome implies that the risk factor of interest may have a causal effect on the outcome [17]. Previous MR analyses on lung cancer risk showed that a genetic score for increased BMI raised the risk for lung cancer, especially for squamous cell carcinoma and small cell lung cancer [18,19]. These results are in contradiction to observational findings, confirming the utility of MR analyses for this exposure.

The goal of the current study was to use genetic variations associated with a range of metabolic factors, including obesity, body shape, dyslipidemia and hyperglycemia, to further investigate the causal relationship between these metabolic exposures and lung cancer. The method applied in this study is called two-sample MR, which combines summary statistics on genetic variant-exposure and genetic variant-outcome associations from different samples [20,21]. Furthermore, we sought to confirm that the genetic control of these metabolic phenotypes did not influence cigarette smoking behavior.

## Methods

### Genetic instruments for obesity and metabolic parameters

Genetic instruments for potential risk factors were independent (linkage disequilibrium (LD) R^2^ < 0.01 in European 1000 Genomes Phase3 samples [22]) single nucleotide polymorphisms (SNPs) that were associated with the trait of interest in the most recent genome-wide association study (GWAS) (P<5x10^-8^) on European ancestry samples. Results from the Genetic Investigation of ANthropometric Traits (GIANT) consortium were used to identify genetic proxies for body mass index (BMI) [23], and waist to hip ratio [24]. High-density and low-density lipoprotein cholesterol (HDL and LDL), total cholesterol and triglycerides were selected as lipid profile components. Genetic loci influencing bloodstream levels of these lipids were identified from GWA data provided by the Global Lipids Genetic Consortium (GLGC) [25]. Additionally, genetic instruments of low frequency were also identified for HDL, LDL cholesterol and triglycerides [26]. Finally, hyperglycemia and hyperinsulinemia parameters were reported within the Meta-Analysis of Glucose and Insulin related traits Consortium (MAGIC) at fasting levels and during an oral glucose tolerance test (OGTT). Genetic instruments were identified for plasma levels of fasting glucose, fasting insulin and 2-hour post-challenge glucose [27]. For these metabolic parameters, in order to reduce potential pleiotropic effect from BMI, SNPs that were not associated with the phenotype once adjusted for BMI (P>0.05) were excluded from our analyses. For each identified SNP, the reported effect was for the increasing trait allele expressed in standard deviations (SD) of the trait per-allele (β_GP_) along with the standard error (SE_GP_). For the studies in which the genetic effects were not originally reported in SDs of the trait, they were recalibrated according to the mean SD, weighted for sample size, reported across different case-control samples. SNPs with ambiguous strand codification (A/T or C/G) were replaced by SNPs in tight genetic linkage (R^2^>0.8) using the *proxysnps* R package (European populations) (R Project) or removed from the analyses. Details on i) the number of SNPs identified for each potential risk trait, ii) the mean and SD of the traits in the discovery GWAS, and iii) the proportion of trait variance explained by the genetic proxies are presented in Table 1.

**Table 1:** Number of identified instrumental SNPs for metabolic factors, phenotype distribution in the discovery sample, and proportion of phenotype variance explained by the instruments. SD: standard deviation.

Phenotype	N SNP	Mean ± SD	Units	Variance explained (%)	Consortium	Publication for instruments
Body mass index	73	27.0 ± 4.6	kg/m^2^	2.7	GIANT	Locke et al 2015 [23]
Waist to hip ratio	32	1.1 ± 0.1	cm/cm	1.4	GIANT	Shungin et al 2015 [24]
High density cholesterol	63	53.3 ± 15.5	mg/dl	13.7	GLGC	Willer et al 2013 [25]
Low density cholesterol	50	133.6 ± 38.0	mg/dl	14.6	GLGC	Willer et al 2013 [25]
Total cholesterol	67	213.28 ± 42.6	mg/dl	15.0	GLGC	Willer et al 2013 [25]
Triglycerides	39	140.85 ± 87.8	mg/dl	11.7	GLGC	Willer et al 2013 [25]
HDL rare variants	11	1.4 ± 0.4	mmol/l	4.1	-	Helgadottir et al 2016 [26]
Non-HDL rare variants	11	4.0 ± 1.2	mmol/l	4.1	-	Helgadottir et al 2016 [26]
Triglycerides rare variants	7	1.3 ± (0.8–2.2)	mmol/l	2.4	-	Helgadottir et al 2016 [26]
Fasting glucose	25	5.2 ± 0.8	mmol/l	4.8	MAGIC	Scott et al 2012 [27]
Fasting insulin	11	56.9 ± 44.4	pmol/l	1.2	MAGIC	Scott et al 2012 [27]
2h post-challenge glucose	6	5.6 ± 1.7	mmol/l	1.7	MAGIC	Scott et al 2012 [27]

### Lung cancer association results

GWAS results on overall lung cancer risk and by histology type were obtained from the recently completed meta-analyses [28] comprising the previous TRICL-ILCCO lung cancer GWAS (11,177 lung cancer cases and 40,396 controls) [29] and an additional 18,089 lung cancer samples and 16,054 controls genotyped using the Illumina Infinium OncoArray-500K BeadChip (Illumina Inc. San Diego, CA). The overall sample size was 29,266 lung cancer cases and 56,450 controls. GWAS analyses were stratified by histology, including 11,273 adenocarcinomas, 7,426 squamous cell carcinomas and 2,664 small cell lung cancers. Additionally, analyses were performed stratified by smoking status defined as ever smokers (current and former smokers; 23,223 cases and 16,964 controls) and never smokers (2,355 cases and 7,504 controls). SNPs with imputation quality score (R^2^) less than 0.7 were removed from the datasets. For all SNPs used as instruments in this report, SNP to phenotype effect (β_GP_) and SNP to disease effect (β_GD_) for each lung cancer subgroup can be observed in S1 Table.

### Power assessment

Power calculations on MR analyses were performed according to Burgess [30], given the number of cases and controls of the lung cancer subgroups, for five genetic instruments accounting for different proportion of explained variance (1%, 2.5%, 5%, 10% and 15%) within the range of our genetic instrument sets (see Table 1). Power calculations are presented in S1 Fig.

### Mendelian randomization analyses on lung cancer

The causal risk effects of metabolic factors on lung cancer overall, as well as by histology and other subgroups were estimated using a likelihood-based approach, which is considered the most accurate method to estimate causal effects when there is a continuous log-linear association between risk factor and disease risk [20]. The resulting odds ratio (OR) and 95% confidence interval (CI) provide an estimate of relative risk caused by each SD increase in the exposure. We investigated the heterogeneity of causal effects by histology and by smoking status, estimating the *I*^*2*^ statistic assuming a fixed-effect model using the *meta* R package (R project). We also provide OR estimates using the complementary weighted median method wherein the causal effect estimate is weighted towards the median of the distribution of instrumental SNPs [31]. This approach is less sensitive to individual instrumental SNPs behaving as outliers. Furthermore, because OR estimates can be confounded if the instrumental genetic variants are also associated with other causal risk factors, i.e. through directional pleiotropy, we also performed MR-Egger [32]. This approach involves a weighted linear regression of the SNP to disease effects (β_GD_) on the SNP to phenotype effects (β_GP_), and provides a direct estimate of any directional pleiotropy under the assumption that pleiotropic biases affecting the overall MR risk estimates are acting in the same direction [33]. Additionally, MR-Egger regression is able to identify SNPs behaving as genetic outliers, providing Bonferroni corrected P-values. We also provide a funnel plot of the fold risk increase (exp(β_GD_/β_GP_)) vs. the instrumental strength of each SNP (β_GP_/SE_GD_^2^) to allow visual assessment of asymmetry of instrumental causal estimates. These plots were generated using the *ggplot2* R package (R Project).

### Assessment of association between the genetic instruments and other phenotypes

To evaluate whether MR lung cancer risk estimates could be generated by other phenotypes than the instrumented ones, the association of genetic instruments with other phenotypes was assessed. The effects of our genetic instruments on cigarette smoking was evaluated using a novel online platform for Mendelian randomization based on summary genetic association data from GWAS (MR-Base; http://www.mrbase.org/) [34]. Genetic effects on cigarette smoking (cigarettes per day) were obtained from the Tobacco and Genetics (TAG) consortium [35]. TAG data were generated from 16 independent studies which included 38,181 smokers with information on number of smoked cigarettes per day. We also assessed the association of dyslipidemia and hyperglycemia genetic instruments with BMI using data from the GIANT consortium [23].

## Results

### Obesity and body shape parameters

**Fig 1:**
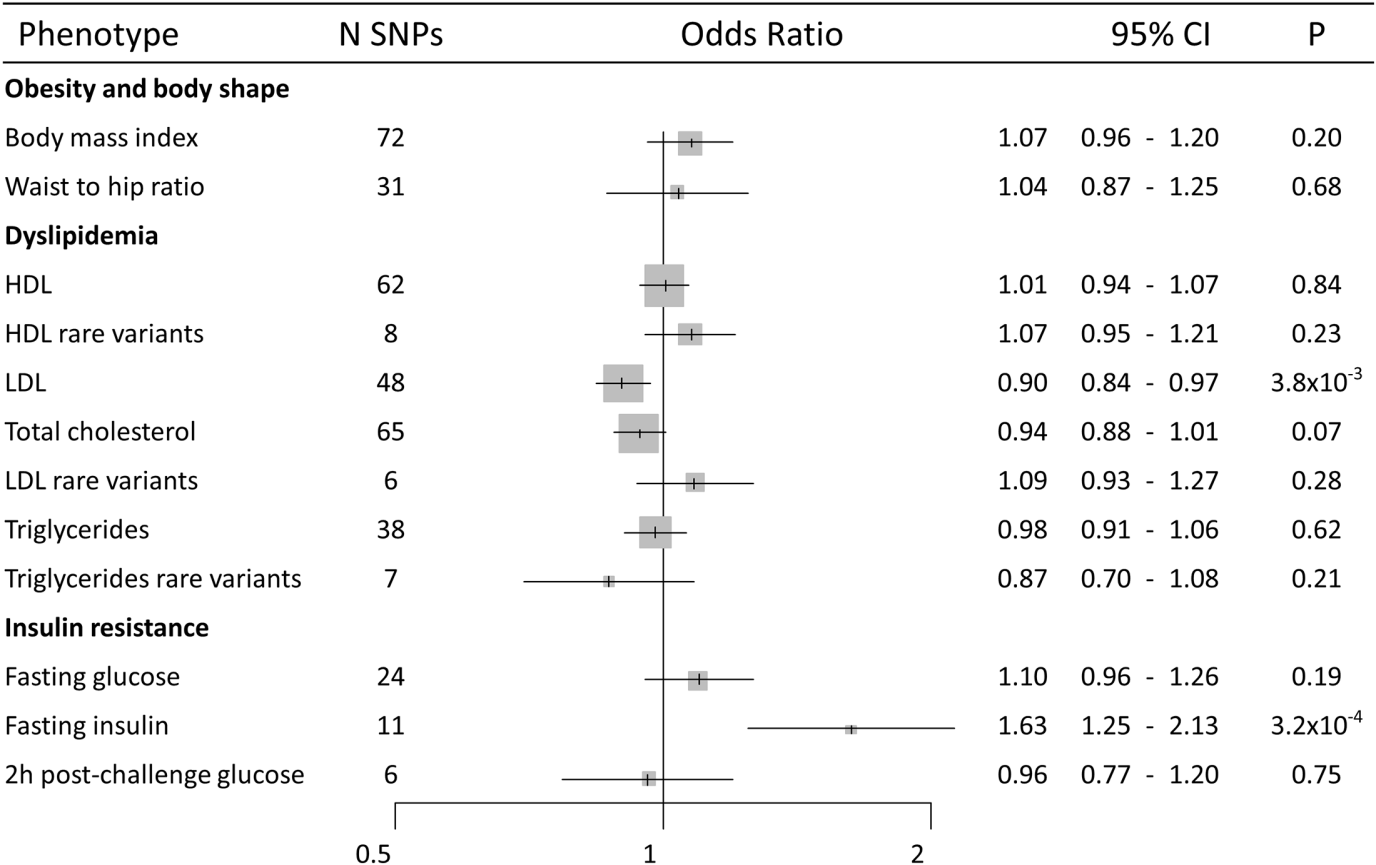
Forest plot of lung cancer overall risk for one standard deviation (Table 1) increase in each phenotype provided by the MR likelihood-based approach. CI: Confidence interval. P: P value.

**Fig 2:**
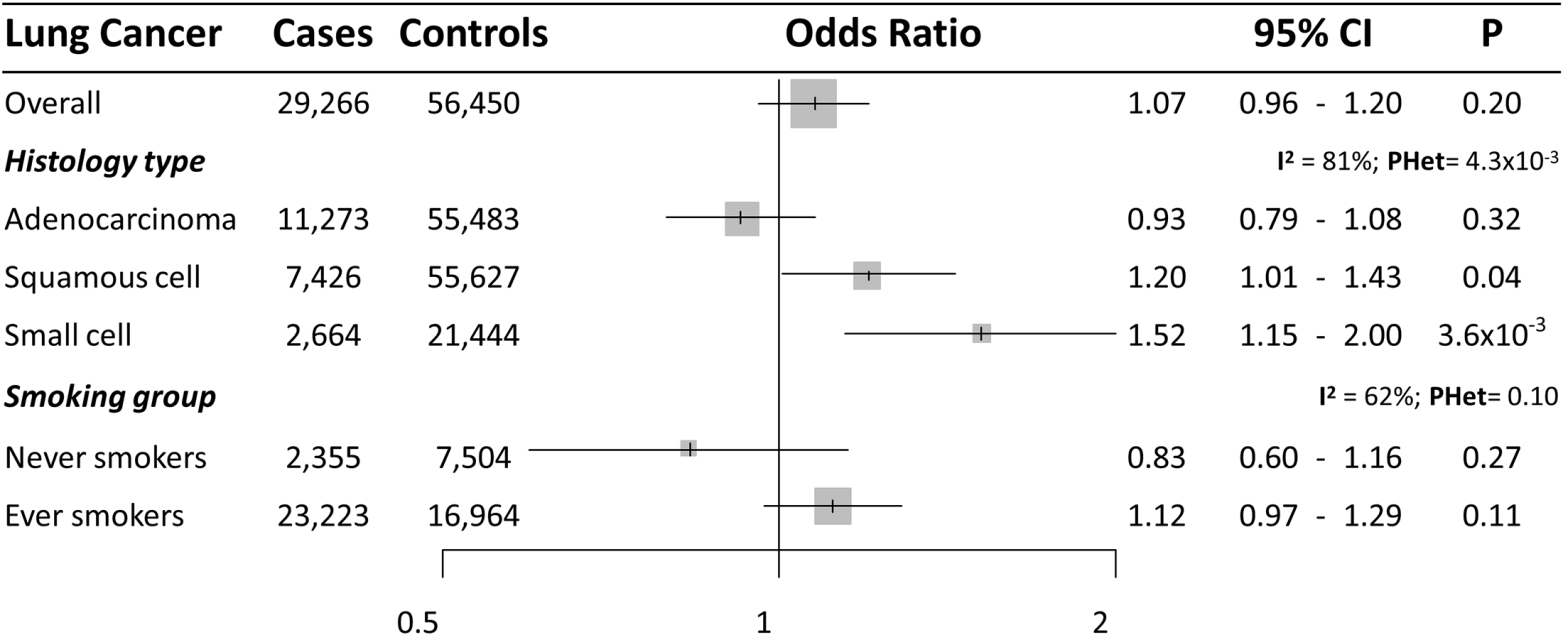
Forest plot of lung cancer risk for each SD increase in BMI (approximately 4.6 kg/m^2^) observed in the likelihood-based MR approach. 95%CI: 95% Confidence Interval; P: P value. I^2^: between-strata heterogeneity. PHet: P value of between-strata heterogeneity.

**Fig 3:**
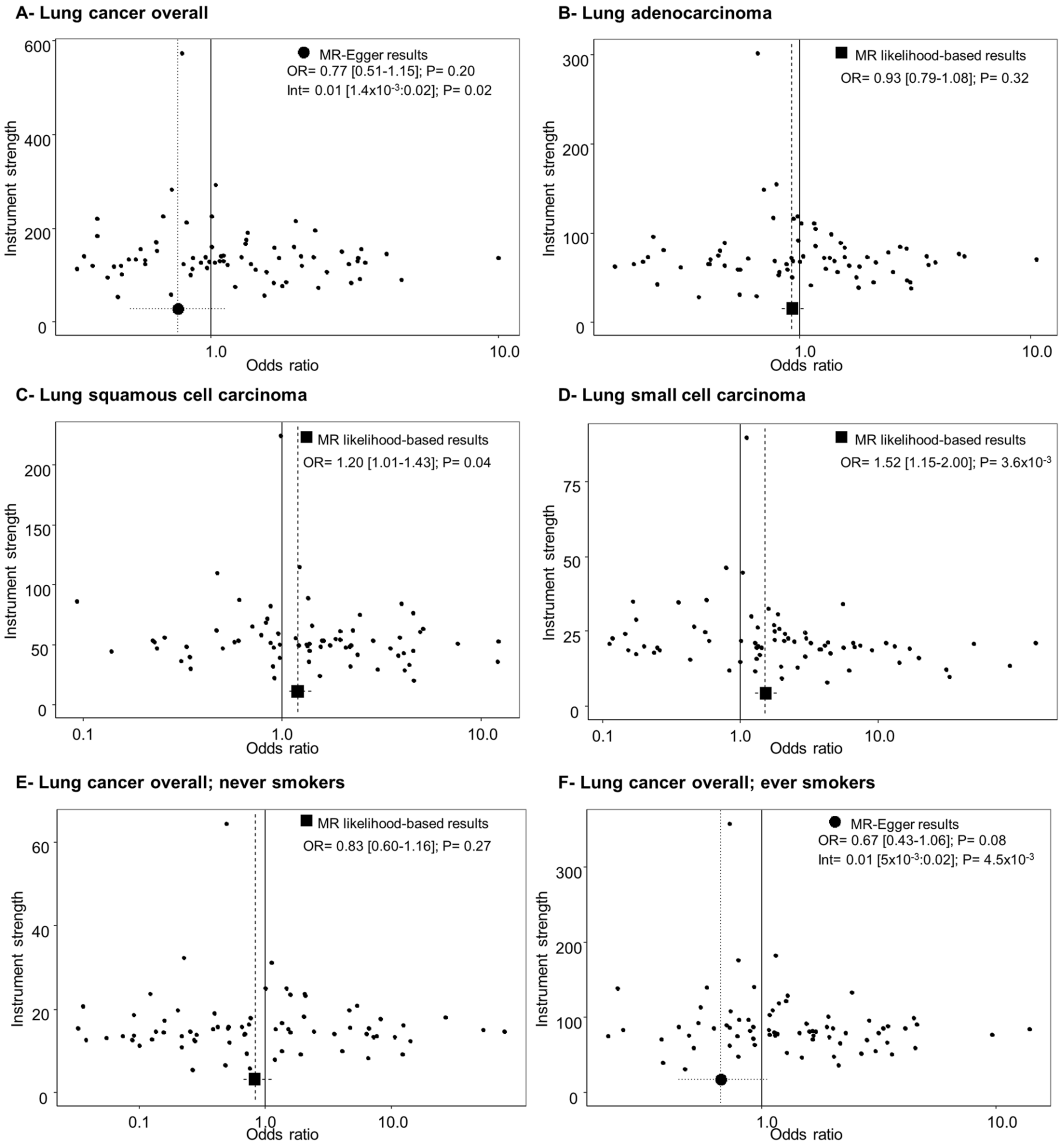
Funnel plots for the distribution of risk estimates of BMI instrumental SNPs along with MR causal effect lung cancer subtypes. OR: Odds ratio; Int: Intercept; P: P value.

Based on 72 SNPs used as genetic instruments for BMI, we estimated an overall OR (per SD increase in BMI (4.6 kg/m^2^)) of 1.07 (95%CI = 0.96–1.20) for lung cancer risk (Fig 1). However, stratified analyses by histology subtypes revealed heterogeneity in the causal effect estimates (*P*_*heterogeneity*_ *= 4*.*3x10*^*-3*^), with no effect on adenocarcinoma (OR [95%CI] = 0.93 [0.79–1.08]), but an increased risk for squamous cell carcinoma (OR [95%CI] = 1.20 [1.01–1.43]) and small cell lung cancer (OR [95%CI] = 1.52 [1.15–2.00]) (Fig 2). Analyses stratified by smoking status showed a null effect of BMI in never smokers (OR [95%CI] = 0.83 [0.60–1.16]) and in ever smokers (OR [95%CI] = 1.12 [0.97–1.29]) (*P*_*heterogeneity*_ = 0.10) (Fig 2). Weighted median MR approach provided similar risk estimates for the different lung cancer types (S2 Table). The MR-Egger intercept test revealed the presence of bias on the initial risk estimation on lung cancer overall and in ever-smokers (S3 Table), with MR-Egger risk estimates indicating no effect of BMI on lung cancer overall. Potential asymmetry of the SNP’ risk estimates reflected the presence of directional pleiotropy for lung cancer overall (Fig 3A). Funnel plots for the other histological types can be observed in the funnel plots included in Fig 3B–3F. Regarding waist-to-hip ratio, there was no evidence of causal effect on lung cancer overall (OR [95%CI] = 1.04 [0.87–1.25]) (Fig 1) or histology subgroups (S2 Fig). Sensitivity analyses did not detect any effect biasing the initial risk estimates (S2 and S3 Tables). Funnel plots can be observed in S3 Fig.

### Blood lipid levels

**Fig 4:**
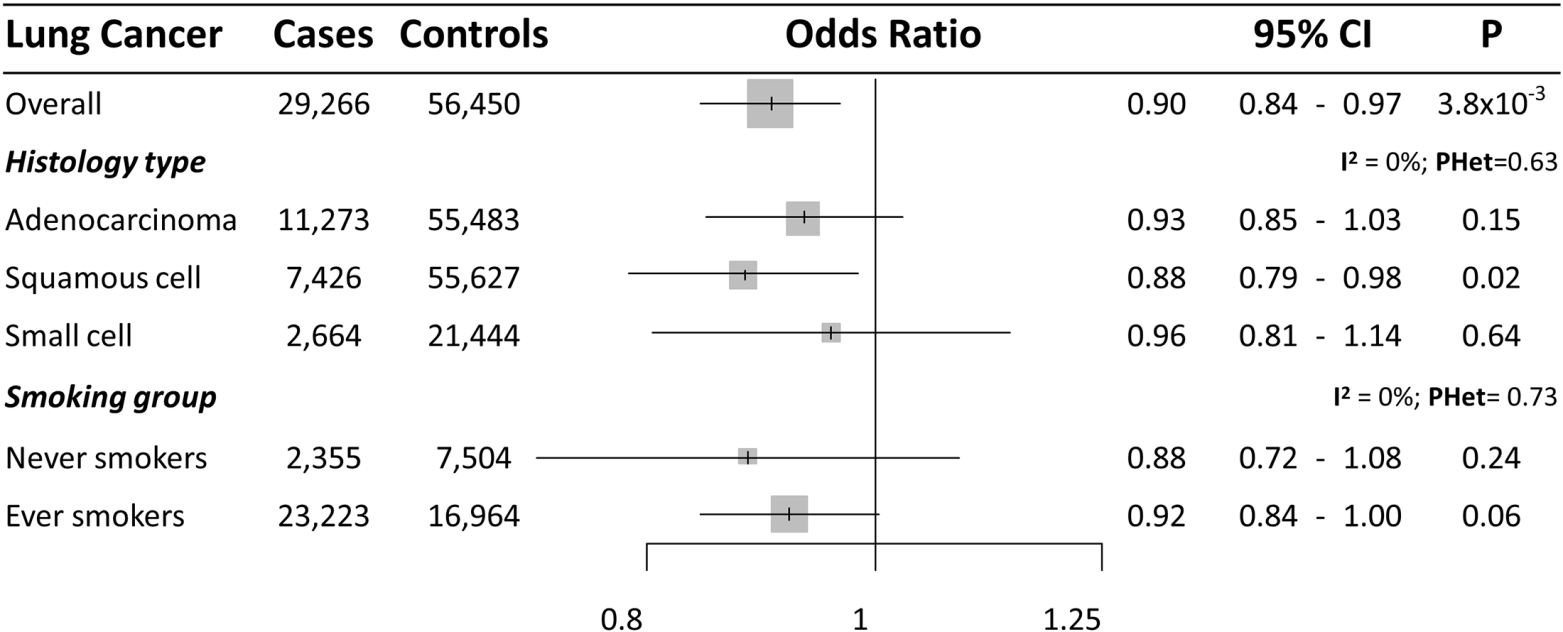
Forest plot of lung cancer risk for each SD increase in LDL (approximately 38.0 mg/dl) observed in the likelihood-based MR approach using the instrument set of common SNPs. 95%CI: 95% Confidence Interval; P: P value. I^2^: between-strata heterogeneity. PHet: P value of between-strata heterogeneity.

There was no consistent evidence of a causal effect of HDL on lung cancer overall (OR [95%CI] = 1.01 [0.94–1.07] for instrumental common SNPs, and OR [95%CI] = 1.07 [0.95–1.21] for instrumental rare SNPs) (Fig 1), nor any lung cancer subgroup (S4–S7 Figs). Triglyceride levels did not indicate any effect on lung cancer overall; (OR [95%CI] = 0.98 [0.91–1.06] for instrumental common SNPs and OR [95%CI] = 0.87 [0.70–1.08] for instrumental rare SNPs) (Fig 1), or for lung cancer subtypes (S8–S11 Figs). An inverse relationship was observed with overall lung cancer risk for each SD increase in LDL (approximately 38.0 mg/dl) (OR [95%CI] = 0.90 [0.84–0.97] for instrumental common SNPs (Fig 1), although stratified analyses did not provided a consistent inverse association among histology types (OR of 0.93 for adenocarcinoma (95%CI = 0.85–1.03), 0.88 for squamous cell carcinoma (95%CI = 0.79–0.98), and 0.96 for small cell lung cancer (95%CI = 0.81–1.14) (Fig 4 and S12 Fig for funnel plots). Additionally, LDL instrumental rare SNPs did not provide evidence of association with lung cancer overall (OR [95%CI] = 1.09 [0.93–1.27]) (Fig 1) or subtypes (S13 and S14 Figs for funnel plots). Finally, total cholesterol showed a similar pattern of association with lung cancer overall and with subtypes that LDL common SNPs (OR of 0.94 for lung cancer overall (95%CI = 0.88–1.01), OR of 1.01 for adenocarcinoma (95%CI = 0.92–1.10), 0.89 for squamous cell carcinoma (95%CI = 0.80–1.00), and 0.93 for small cell lung cancer (95%CI = 0.79–1.10)) (S15 and S16 Figs for funnel plots).

### Insulin resistance parameters

**Fig 5:**
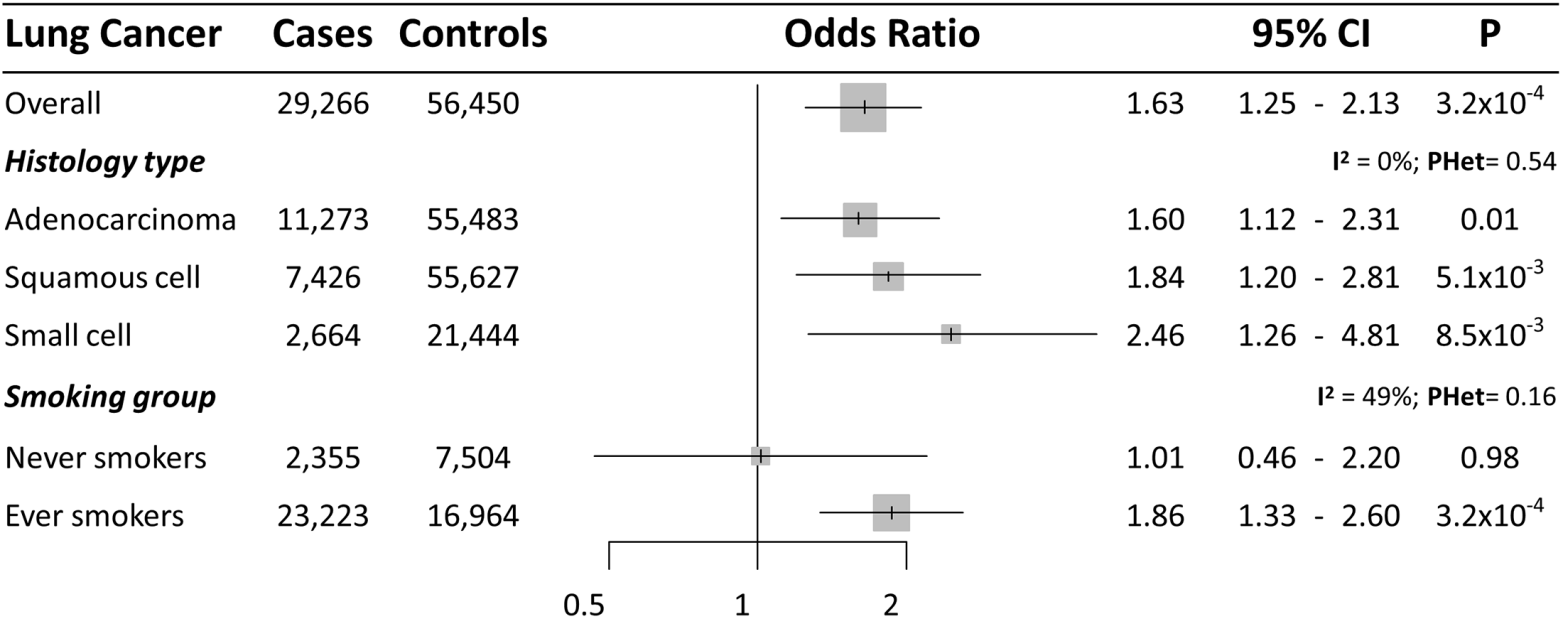
Forest plot of lung cancer risk for each SD increase in fasting insulin (approximately 44.4 pmol/L) observed in the likelihood-based MR approach. 95%CI: 95% Confidence Interval; P: P value. I^2^: between-strata heterogeneity. PHet: P value of between-strata heterogeneity.

Among glucose and insulin parameters, fasting insulin was associated with an increased risk in overall lung cancer (OR [95%CI] = 1.63 [1.25–2.13] per each SD increase [44.4 pmol/l]) (Fig 1). Stratified analyses provided consistent associations with all histology subtypes (Fig 5), with a risk increase of 1.60 for adenocarcinoma (95%CI = 1.12–2.31), 1.84 for squamous cell carcinoma (95%CI = 1.20–2.81), and 2.46 for small cell lung cancer (95%CI = 1.26–4.81). Stratified analyses by smoking status showed some heterogeneity in causal effects (*P*_*heterogeneity*_ *= 0*.*16)* (Fig 5), with a clear increased risk among ever smokers (OR [95%CI] = 1.86 [1.33–2.60]), but no association among never smokers (OR [95%CI] = 1.01 [0.46–2.20]). Sensitivity tests identified rs4865796 as an outlier SNP in the analyses for lung adenocarcinoma subtype and lung cancer overall in ever smokers. Removing this SNP, the analyses indicated slightly higher levels of risk associated with this genetic instrument (Funnel plots in S17 Fig).

Fasting glucose showed little evidence for an association with overall lung cancer risk (OR per SD of 0.8 mmol/l = 1.10; 95%CI = 0.96–1.26) (Fig 1), nor for adenocarcinoma (OR [95%CI] = 1.03 [0.85–1.24] or small cell lung cancer (OR [95%CI] = 1.19 [0.83–1.68]), where a positive association with risk was seen for squamous cell carcinoma (OR [95%CI] = 1.32 [1.06–1.65]) (S18 and S19 Figs for funnel plots). Finally, 2-hour post-challenge glucose showed no evidence of causality for lung cancer overall (OR [95%CI] = 0.96 [0.77–1.20]) (Fig 1) or any lung cancer subgroup (S20 and S21 Figs for funnel plots).

### Assessment of association between the genetic instruments and other phenotypes

Using MR-Base, we also evaluated the association between cigarette smoking for each genetic instrument associated with lung cancer risk, including BMI, LDL, and fasting insulin. Each SD increase in BMI increased cigarette consumption by 1.27 cigarettes (95%CI = 0.46:2.07; P = 2.1x10^-3^; MR likelihood-based approach), while the other metabolic genetic instruments were not associated with cigarette smoking (MR likelihood-based estimate per SD increase [95%CI] = -0.35 [-0.92:0.22] for LDL and 1.21 [-0.67:3.08] for fasting insulin). Additionally, each SD increase in LDL was inversely associated with BMI (-0.02 SD in BMI (95%CI = -0.04:-3.8x10^-3^; P = 0.02), while each SD increase in fasting was not associated with BMI (0.07 SD in BMI (95%CI = -0.01:0.15; P = 0.10)).

## Discussion

We have used existing data from large GWAS on metabolic factors to identify potentially causal risk factors of lung cancer that are amenable to intervention. Our results suggested that higher BMI is associated with an increase in risk of squamous and small cell carcinoma of the lung, and that this relation may be mediated by altered smoking behavior. Additionally, we found consistent evidence of a positive association between fasting insulin and overall lung cancer risk, as well as an inverse relationship between LDL levels and lung cancer risk.

Obesity and related metabolic factors are associated with risk of several cancers [36], but traditional observational studies are not reliable for assessing the association between metabolic factors and cancers strongly associated with smoking. The strength of the association between BMI and lung cancer subtypes found in this study, were weaker than those reported in previous MR analyses (45%-81% of risk increase) [18,19]. These previous studies were based on smaller sample sizes (12,160 and 16,572 lung cancer cases, respectively), whereas our current study provides causal estimates from 29,266 lung cancer cases and 56,450 controls, including the samples of previous studies. Our analysis also detected a strong positive effect of each unit increase in BMI on number of cigarettes smoked per day, indicating that the associations with risk of squamous and small cell carcinoma lung cancer are caused by an influence of BMI on smoking behavior. Given that this association was derived from genetic data on BMI, and therefore not influenced by any inverse effect that tobacco consumption may have on BMI levels, it has important implications beyond this study on the smoking behavior and smoking prevention.

Our results lend further support to epidemiological studies showing an increased lung cancer risk for the effect of reduced lipid levels [9] and elevated circulating insulin [11]. Several studies have reported an inverse relationship between total cholesterol levels and cancer risk [37], which has been previously interpreted as an effect of preclinical cancer due to an increased uptake of cholesterol by tumor cells [9]. In contrast, our study suggests a potential relationship between cholesterol levels and lung cancer. However, cholesterol genetic instruments using common and rare SNPs did not provide homogeneous results. This could be explained by different observations: by the inverse relationship between the instrument set of common SNPs for cholesterol and BMI revealed by a sensitivity analysis using MR-Base, by a multiple testing effect, but also by the different proportion of exposure that genetic instruments represent (instrument sets of rare SNPs were weaker and had less power to detect potential causal associations). Further MR mediation analyses are needed in order to clarify the causal relationship between lipid levels and lung cancer accounting for the potential effect of BMI and smoking. Finally, the risk increase observed by fasting glucose and fasting insulin is unlikely to be driven by cigarette smoking or BMI, since the relevant instrumental sets are not associated with smoking or BMI. A potential causal relationship between increased insulin and cancer risk using genetic instruments has also been reported for endometrial cancer [38] and pancreatic cancer [39]. These results lend further support for an important role of the insulin-pathway in cancer aetiology [40–42].

One of the main caveats in Mendelian randomization studies is the potential violation of methodological assumptions, in particular the lack of directional pleiotropic effects biasing association estimates. In the current study we evaluated the extent to which pleiotropy may have affected our risk estimates using MR-Egger regression, as well as the MR-Base platform to directly assess the relation between genetic instruments and potential confounders.

In conclusion, using a two-sample Mendelian randomization approach, this study assessed a range of metabolic factors in relation to lung cancer risk. Our results revealed that genetic associations between BMI and lung cancer subtypes could be mediated by a positive relationship between BMI and cigarette consumption. Our results are also consistent with observational studies that indicate an inverse relationship between lipid levels and lung cancer risk, and an increasing risk caused by elevated fasting insulin levels.

## Supporting information

S1 Fig Power calculations for Mendelian randomization analyses on lung cancer using genetic instruments accounting for different proportion of phenotypic variance (15.0, 10.0, 5.0, 2.5, and 1.0%). (PDF)

S2 Fig Forest plot of lung cancer risk for each SD increase of waist-to-hip ratio observed in a likelihood-based MR approach. 95%CI: 95% Confidence Interval; P: P value. I^2^: between-strata heterogeneity. PHet: P value of between-strata heterogeneity.
(PDF)

S3 Fig Funnel plots for the distribution of risk estimates of waist-to-hip ratio-instrumental SNPs along with MR causal effect lung cancer subtypes. OR: Odds ratio; Int: Intercept; P: P value.
(PDF)

S4 Fig Forest plot of lung cancer risk for each SD increase in HDL observed in a likelihood-based MR approach using the instrument set of common SNPs. 95%CI: 95% Confidence Interval; P: P value. I^2^: between-strata heterogeneity. PHet: P value of between-strata heterogeneity.
(PDF)

S5 Fig Forest plot of lung cancer risk for each SD increase in HDL observed in a likelihood-based MR approach using the instrument set of rare SNPs. 95%CI: 95% Confidence Interval; P: P value. I^2^: between-strata heterogeneity. PHet: P value of between-strata heterogeneity.
(PDF)

S6 Fig Funnel plots for the distribution of risk estimates of HDL instrumental common SNPs along with MR causal effect lung cancer subtypes. OR: Odds ratio; Int: Intercept; P: P value.
(PDF)

S7 Fig Funnel plots for the distribution of risk estimates of HDL instrumental rare SNPs along with MR causal effect lung cancer subtypes. OR: Odds ratio; Int: Intercept; P: P value.
(PDF)

S8 Fig Forest plot of lung cancer risk for each SD increase in triglycerides observed in a likelihood-based MR approach using the main instrument set of common SNPs. 95%CI: 95% Confidence Interval; P: P value. I^2^: between-strata heterogeneity. PHet: P value of between-strata heterogeneity.
(PDF)

S9 Fig Forest plot of lung cancer risk for each SD increase in triglycerides observed in a likelihood-based MR approach using the instrument set of rare SNPs. 95%CI: 95% Confidence Interval; P: P value. I^2^: between-strata heterogeneity. PHet: P value of between-strata heterogeneity.
(PDF)

S10 Fig Funnel plots for the distribution of risk estimates of triglycerides instrumental common SNPs along with MR causal effect lung cancer subtypes. OR: Odds ratio; Int: Intercept; P: P value.
(PDF)

S11 Fig Funnel plots for the distribution of risk estimates of triglycerides instrumental rare SNPs along with MR causal effect lung cancer subtypes. OR: Odds ratio; Int: Intercept; P: P value.
(PDF)

S12 Fig Funnel plots for the distribution of risk estimates of LDL instrumental common SNPs along with MR causal effect lung cancer subtypes. OR: Odds ratio; Int: Intercept; P: P value.
(PDF)

S13 Fig Forest plot of lung cancer risk for each SD increase in LDL observed in a likelihood-based MR approach using the instrument set of rare SNPs. 95%CI: 95% Confidence Interval; P: P value. I^2^: between-strata heterogeneity. PHet: P value of between-strata heterogeneity.
(PDF)

S14 Fig Funnel plots for the distribution of risk estimates of LDL instrumental rare SNPs along with MR causal effect lung cancer subtypes. OR: Odds ratio; Int: Intercept; P: P value.
(PDF)

S15 Fig Forest plot of lung cancer risk for each SD increase in total cholesterol observed in a likelihood-based MR approach. 95%CI: 95% Confidence Interval; P: P value. I^2^: between-strata heterogeneity. PHet: P value of between-strata heterogeneity.
(PDF)

S16 Fig Funnel plots for the distribution of risk estimates of total cholesterol instrumental SNPs along with MR causal effect lung cancer subtypes. OR: Odds ratio; Int: Intercept; P: P value.
(PDF)

S17 Fig Funnel plots for the distribution of risk estimates of fasting insulin instrumental SNPs along with MR causal effect lung cancer subtypes. OR: Odds ratio; Int: Intercept; P: P value.
(PDF)

S18 Fig Forest plot of lung cancer risk for each SD increase in fasting glucose observed in a likelihood-based MR approach. 95%CI: 95% Confidence Interval; P: P value. I^2^: between-strata heterogeneity. PHet: P value of between-strata heterogeneity.
(PDF)

S19 Fig Funnel plots for the distribution of risk estimates of fasting glucose instrumental SNPs along with MR causal effect lung cancer subtypes. OR: Odds ratio; Int: Intercept; P: P value.
(PDF)

S20 Fig Forest plot of lung cancer risk for each SD increase in glucose 2h post-challenge observed in a likelihood-based MR approach. 95%CI: 95% Confidence Interval; P: P value. I^2^: between-strata heterogeneity. PHet: P value of between-strata heterogeneity.
(PDF)

S21 Fig Funnel plots for the distribution of risk estimates of glucose 2h post-challenge instrumental SNPs along with MR causal effect lung cancer subtypes. OR: Odds ratio; Int: Intercept; P: P value.
(PDF)

S1 Table Association parameters of instrumental SNPs for the corresponding metabolic factor and for different lung cancer groups. CHR: Chromosome. BP: Base pair. SE: Standard error. BMI: Body mass index. HDL: High-density lipoprotein, LDL: Low-density lipoprotein.
(PDF)

S2 Table Risk increase on lung cancer phenotypes for each standard deviation increase in the phenotype provided by weighted median MR approach. HDL: High-density lipoprotein, LDL: Low-density lipoprotein. Chol: Cholesterol. OR: Odds ratio. LCI: Lower confidence interval. UCI: Upper confidence interval. P: P value.
(PDF)

S3 Table Overall pleiotropic effect assessment of causal estimates of potential risk factors on lung cancer phenotypes provided by MR-Egger test. Int: Intercept. HDL: High-density lipoprotein, LDL: Low-density lipoprotein. Chol: Cholesterol. OR: Odds ratio. Est: Estimate. LCI: Lower confidence interval. UCI: Upper confidence interval. P: P value.
(PDF)
