# Supplementary material for: Obesity, metabolic factors and risk of different histological types of lung cancer: A Mendelian randomization study
Source: PLoS One. 2017 Jun 8;12(6):e0177875. doi: 10.1371/journal.pone.0177875 (PMC5464539; doi:10.1371/journal.pone.0177875)

S1 Fig - Power calculations for Mendelian randomization analyses on lung cancer using genetic instruments accounting for different proportion of phenotypic variance (15.0, 10.0, 5.0, 2.5 , and 1.0%).

A- Lung cancer overall

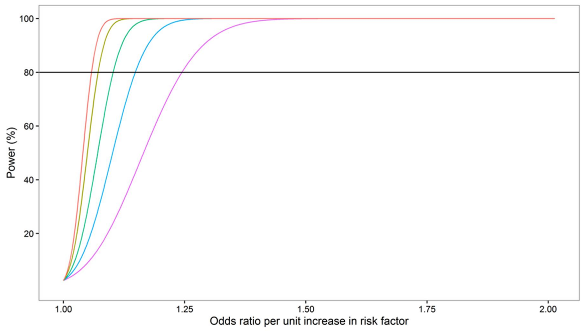

B- Lung adenocarcinoma

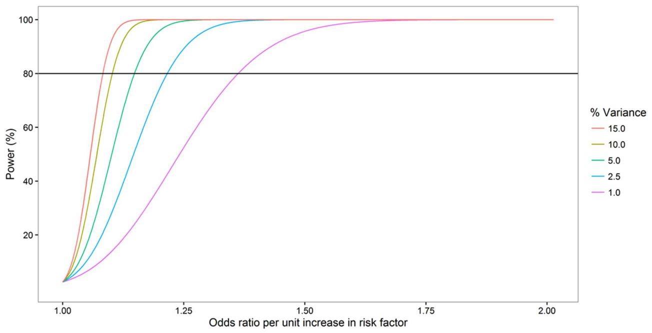

C- Lung squamous cell carcinoma

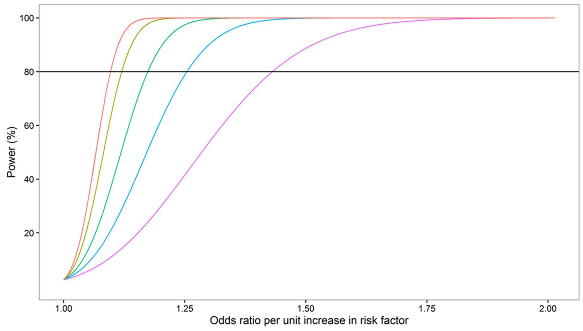

D- Lung small cell carcinoma

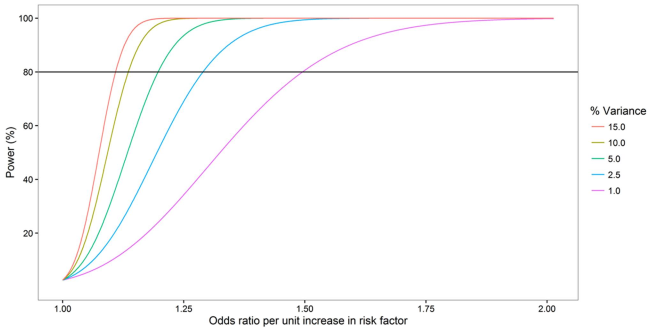

E- Lung cancer overall; never smokers

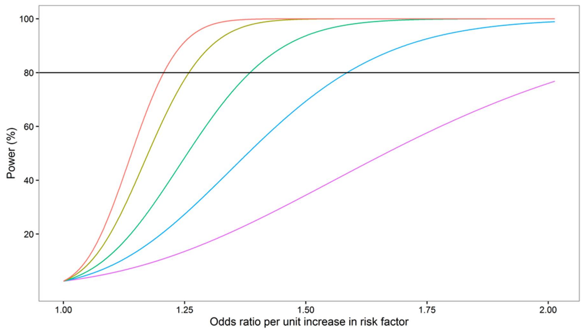

F- Lung cancer overall; ever smokers

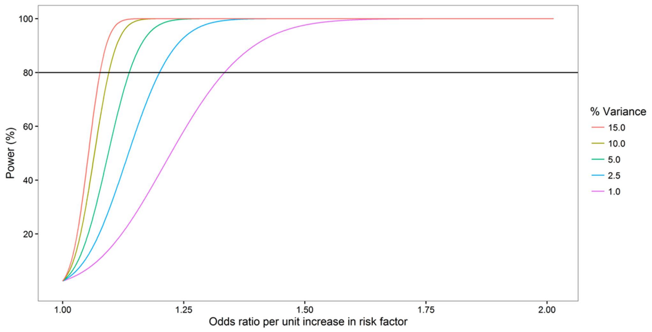

Supplement: S1 Fig — (PDF) [file pone.0177875.s001.pdf]
