# Supplementary material for: Obesity, metabolic factors and risk of different histological types of lung cancer: A Mendelian randomization study
Source: PLoS One. 2017 Jun 8;12(6):e0177875. doi: 10.1371/journal.pone.0177875 (PMC5464539; doi:10.1371/journal.pone.0177875)

S2 Fig - Forest plot of lung cancer risk for each SD increase of waist-to-hip ratio observed in a likelihood-based MR approach. 95%CI: 95% Confidence Interval; P: P value. I^2: between-strata heterogeneity. PHet: P value of between-strata heterogeneity.

Lung Cancer	Cases	Controls	Odds Ratio	95% CI	P
Overall	29,266	56,450	1.04	0.87 - 1.25	0.68
Histology type					**I^2** = 0%; **PHet**= 0.70
Adenocarcinoma	11,273	55,483	0.97	0.76 - 1.25	0.83
Squamous cell	7,426	55,627	1.11	0.83 - 1.49	0.48
Small cell	2,664	21,444	1.17	0.74 - 1.86	0.50
Smoking group					**I^2** = 0%; **PHet**= 0.42
Never smokers	2,355	7,504	0.78	0.45 - 1.35	0.37
Ever smokers	23,223	16,964	1.00	0.79 - 1.26	0.99

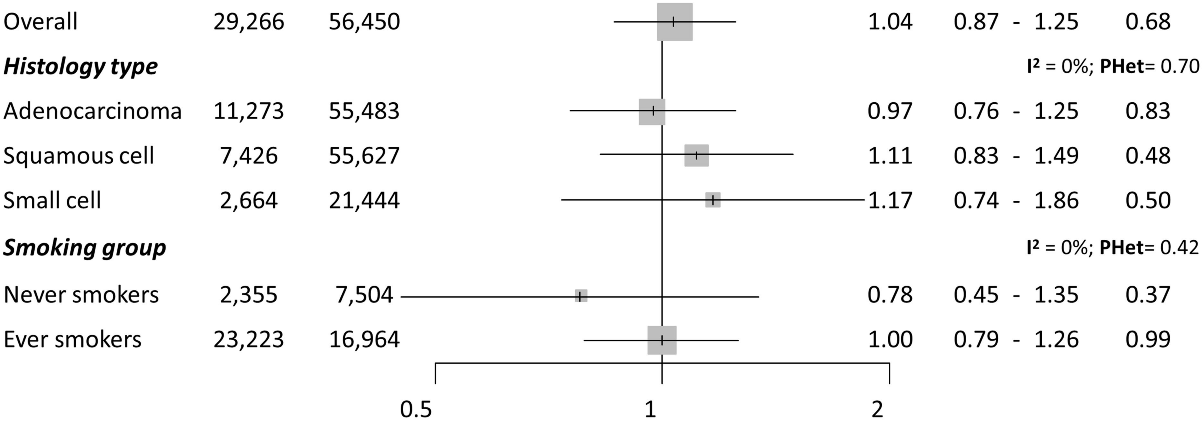

Supplement: S2 Fig — 95%CI: 95% Confidence Interval; P: P value. I2: between-strata heterogeneity. PHet: P value of between-strata heterogeneity. (PDF) [file pone.0177875.s002.pdf]
