# Supplementary material for: Obesity, metabolic factors and risk of different histological types of lung cancer: A Mendelian randomization study
Source: PLoS One. 2017 Jun 8;12(6):e0177875. doi: 10.1371/journal.pone.0177875 (PMC5464539; doi:10.1371/journal.pone.0177875)

S4 Fig - Forest plot of lung cancer risk for each SD increase in HDL observed in a likelihood-based MR approach using the instrument set of common SNPs. 95%CI: 95% Confidence Interval; P: P value. I^2: between-strata heterogeneity. PHet: P value of between-strata heterogeneity.

Lung Cancer	Cases	Controls	Odds Ratio	95% CI	P
Overall	29,266	56,450	1.01	0.94 - 1.07	0.84
Histology type				I^2 = 0%; **PHet**=0.87	
Adenocarcinoma	11,273	55,483	1.01	0.93 - 1.10	0.80
Squamous cell	7,426	55,627	1.02	0.92 - 1.12	0.77
Small cell	2,664	21,444	1.06	0.90 - 1.25	0.47
Smoking group				I^2 = 0%; **PHet**= 0.94	
Never smokers	2,355	7,504	1.02	0.84 - 1.23	0.84
Ever smokers	23,223	16,964	1.03	0.95 - 1.11	0.50

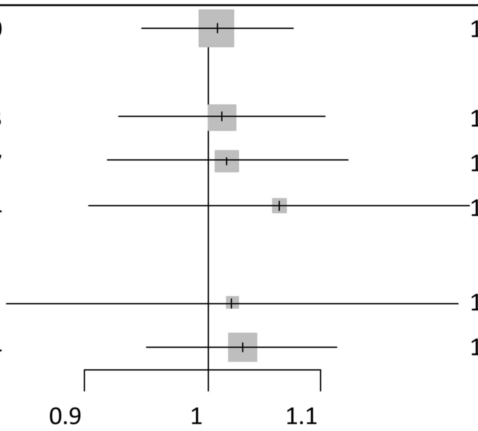

Supplement: S4 Fig — 95%CI: 95% Confidence Interval; P: P value. I2: between-strata heterogeneity. PHet: P value of between-strata heterogeneity. (PDF) [file pone.0177875.s004.pdf]
