# Supplementary material for: Obesity, metabolic factors and risk of different histological types of lung cancer: A Mendelian randomization study
Source: PLoS One. 2017 Jun 8;12(6):e0177875. doi: 10.1371/journal.pone.0177875 (PMC5464539; doi:10.1371/journal.pone.0177875)

S5 Fig - Forest plot of lung cancer risk for each SD increase in HDL observed in a likelihood-based MR approach using the instrument set of rare SNPs. 95%CI: 95% Confidence Interval; P: P value. I^2: between-strata heterogeneity. PHet: P value of between-strata heterogeneity.

Lung Cancer	Cases	Controls	Odds Ratio	95% CI	P
Overall	29,266	56,450	1.07	0.95 - 1.21	0.23
Histology type					I^2 = 78%; **PHet**=0.01
Adenocarcinoma	11,273	55,483	1.03	0.87 - 1.21	0.73
Squamous cell	7,426	55,627	1.36	1.12 - 1.65	2.0×10^{-3}
Small cell	2,664	21,444	0.81	0.60 - 1.10	0.18
Smoking group					I^2 = 0%; **PHet**= 0.54
Never smokers	2,355	7,504	0.88	0.62 - 1.27	0.50
Ever smokers	23,223	16,964	1.00	0.86 - 1.16	0.97

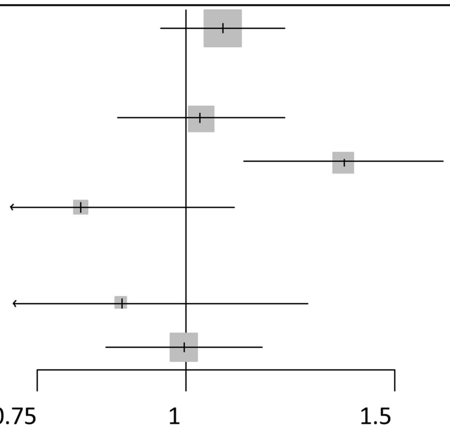

Supplement: S5 Fig — 95%CI: 95% Confidence Interval; P: P value. I2: between-strata heterogeneity. PHet: P value of between-strata heterogeneity. (PDF) [file pone.0177875.s005.pdf]
