# Supplementary material for: Obesity, metabolic factors and risk of different histological types of lung cancer: A Mendelian randomization study
Source: PLoS One. 2017 Jun 8;12(6):e0177875. doi: 10.1371/journal.pone.0177875 (PMC5464539; doi:10.1371/journal.pone.0177875)

S8 Fig - Forest plot of lung cancer risk for each SD increase in triglycerides observed in a likelihood-based MR approach using the main instrument set of common SNPs. 95%CI: 95% Confidence Interval; P: P value. I^2: between-strata heterogeneity. PHet: P value of between-strata heterogeneity.

Lung Cancer	Cases	Controls	Odds Ratio		95% CI	P
Overall	29,266	56,450		0.98	0.91 - 1.06	0.62
Histology type					I^2 = 0%; **PHet**=0.83	
Adenocarcinoma	11,273	55,483		1.03	0.92 - 1.14	0.65
Squamous cell	7,426	55,627		1.00	0.88 - 1.13	0.98
Small cell	2,664	21,444		0.96	0.78 - 1.17	0.66
Smoking group					I^2 = 0%; **PHet**= 0.71	
Never smokers	2,355	7,504		0.99	0.78 - 1.26	0.96
Ever smokers	23,223	16,964		0.95	0.86 - 1.05	0.28

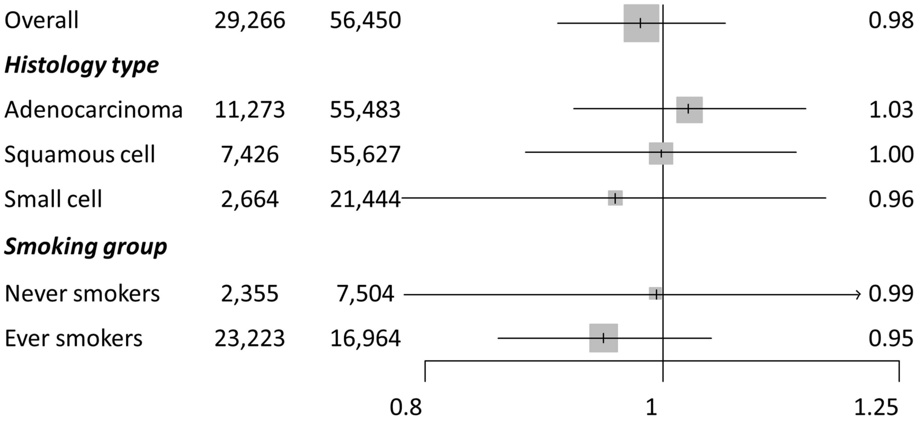

Supplement: S8 Fig — 95%CI: 95% Confidence Interval; P: P value. I2: between-strata heterogeneity. PHet: P value of between-strata heterogeneity. (PDF) [file pone.0177875.s008.pdf]
