# Supplementary material for: Obesity, metabolic factors and risk of different histological types of lung cancer: A Mendelian randomization study
Source: PLoS One. 2017 Jun 8;12(6):e0177875. doi: 10.1371/journal.pone.0177875 (PMC5464539; doi:10.1371/journal.pone.0177875)

S9 Fig - Forest plot of lung cancer risk for each SD increase in triglycerides observed in a likelihood-based MR approach using the instrument set of rare SNPs. 95%CI: 95% Confidence Interval; P: P value. I^2: between-strata heterogeneity. PHet: P value of between-strata heterogeneity.

Lung Cancer	Cases	Controls	Odds Ratio	95% CI	P
Overall	29,266	56,450	0.87	0.70 - 1.08	0.21
Histology type					I^2 = 0%; **PHet**=0.39
Adenocarcinoma	11,273	55,483	0.88	0.66 - 1.18	0.39
Squamous cell	7,426	55,627	0.71	0.50 - 1.01	0.06
Small cell	2,664	21,444	1.10	0.64 - 1.90	0.74
Smoking group					I^2 = 0%; **PHet**= 0.35
Never smokers	2,355	7,504	1.14	0.60 - 2.17	0.68
Ever smokers	23,223	16,964	0.82	0.62 - 1.08	0.16

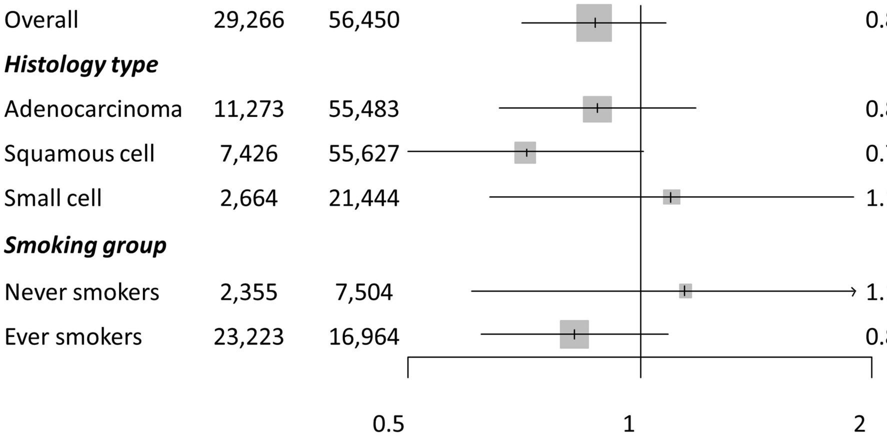

Supplement: S9 Fig — 95%CI: 95% Confidence Interval; P: P value. I2: between-strata heterogeneity. PHet: P value of between-strata heterogeneity. (PDF) [file pone.0177875.s009.pdf]
