# Supplementary material for: Obesity, metabolic factors and risk of different histological types of lung cancer: A Mendelian randomization study
Source: PLoS One. 2017 Jun 8;12(6):e0177875. doi: 10.1371/journal.pone.0177875 (PMC5464539; doi:10.1371/journal.pone.0177875)

S12 Fig - Funnel plots for the distribution of risk estimates of LDL instrumental common SNPs along with MR causal effect lung cancer subtypes. OR: Odds ratio; Int: Intercept; P: P value.

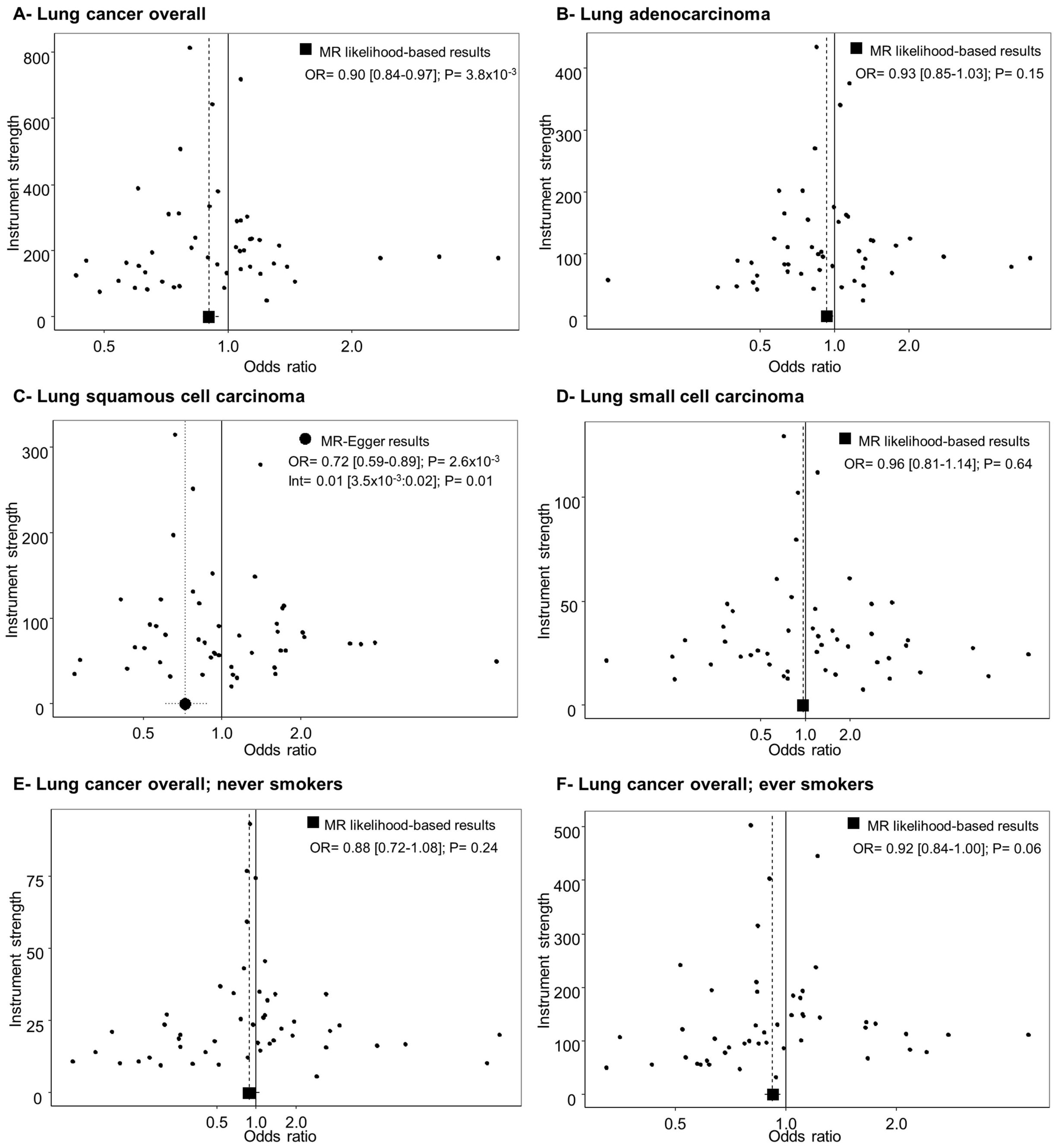

Supplement: S12 Fig — OR: Odds ratio; Int: Intercept; P: P value. (PDF) [file pone.0177875.s012.pdf]
