# Supplementary material for: Obesity, metabolic factors and risk of different histological types of lung cancer: A Mendelian randomization study
Source: PLoS One. 2017 Jun 8;12(6):e0177875. doi: 10.1371/journal.pone.0177875 (PMC5464539; doi:10.1371/journal.pone.0177875)

S13 Fig - Forest plot of lung cancer risk for each SD increase in LDL observed in a likelihood-based MR approach using the instrument set of rare SNPs. 95%CI: 95% Confidence Interval; P: P value. I^2: between-strata heterogeneity. PHet: P value of between-strata heterogeneity.

Lung Cancer	Cases	Controls	Odds Ratio	95% CI	P
Overall	29,266	56,450	1.09	0.93 - 1.27	0.28
Histology type					I^2 = 0%; **PHet**=0.54
Adenocarcinoma	11,273	55,483	1.03	0.83 - 1.26	0.81
Squamous cell	7,426	55,627	1.21	0.95 - 1.55	0.12
Small cell	2,664	21,444	1.01	0.69 - 1.46	0.97
Smoking group					I^2 = 0%; **PHet**= 0.59
Never smokers	2,355	7,504	1.26	0.80 - 1.99	0.32
Ever smokers	23,223	16,964	1.10	0.91 - 1.33	0.32

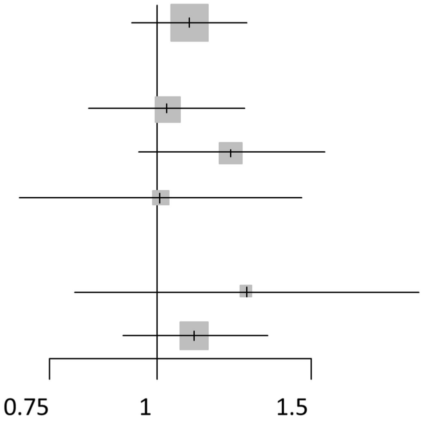

Supplement: S13 Fig — 95%CI: 95% Confidence Interval; P: P value. I2: between-strata heterogeneity. PHet: P value of between-strata heterogeneity. (PDF) [file pone.0177875.s013.pdf]
