# Supplementary material for: Obesity, metabolic factors and risk of different histological types of lung cancer: A Mendelian randomization study
Source: PLoS One. 2017 Jun 8;12(6):e0177875. doi: 10.1371/journal.pone.0177875 (PMC5464539; doi:10.1371/journal.pone.0177875)

S15 Fig - Forest plot of lung cancer risk for each SD increase in total cholesterol observed in a likelihood-based MR approach. 95%CI: 95% Confidence Interval; P: P value. I^2: between-strata heterogeneity. PHet: P value of between-strata heterogeneity.

Lung Cancer	Cases	Controls	Odds Ratio	95% CI	P
Overall	29,266	56,450	0.94	0.88 - 1.01	0.07
Histology type				**I^2** = 30%; **PHet**=0.24	
Adenocarcinoma	11,273	55,483	1.01	0.92 - 1.10	0.86
Squamous cell	7,426	55,627	0.89	0.80 - 1.00	0.04
Small cell	2,664	21,444	0.93	0.79 - 1.10	0.41
Smoking group				**I^2** = 0%; **PHet**= 0.93	
Never smokers	2,355	7,504	0.95	0.78 - 1.16	0.64
Ever smokers	23,223	16,964	0.94	0.87 - 1.03	0.19

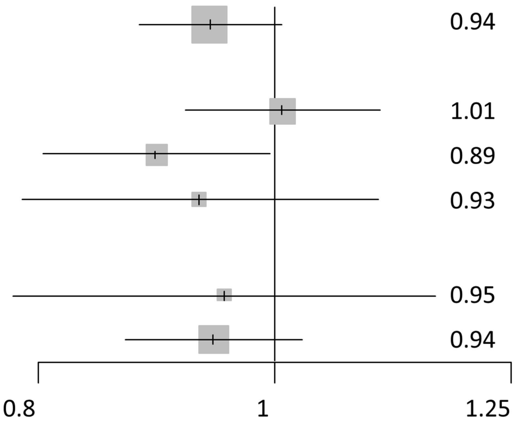

Supplement: S15 Fig — 95%CI: 95% Confidence Interval; P: P value. I2: between-strata heterogeneity. PHet: P value of between-strata heterogeneity. (PDF) [file pone.0177875.s015.pdf]
