# Supplementary material for: Obesity, metabolic factors and risk of different histological types of lung cancer: A Mendelian randomization study
Source: PLoS One. 2017 Jun 8;12(6):e0177875. doi: 10.1371/journal.pone.0177875 (PMC5464539; doi:10.1371/journal.pone.0177875)

S18 Fig - Forest plot of lung cancer risk for each SD increase in fasting glucose observed in a likelihood-based MR approach. 95%CI: 95% Confidence Interval; P: P value. I^2: between-strata heterogeneity. PHet: P value of between-strata heterogeneity.

Lung Cancer	Cases	Controls	Odds Ratio	95% CI	P
Overall	29,266	56,450	1.10	0.96 - 1.26	0.19
Histology type				**I^2** = 31%; **PHet**= 0.24	
Adenocarcinoma	11,273	55,483	1.03	0.85 - 1.24	0.78
Squamous cell	7,426	55,627	1.32	1.06 - 1.65	0.01
Small cell	2,664	21,444	1.19	0.83 - 1.68	0.34
Smoking group				**I^2** = 0%; **PHet**= 0.98	
Never smokers	2,355	7,504	1.11	0.73 - 1.68	0.63
Ever smokers	23,223	16,964	1.11	0.93 - 1.33	0.23

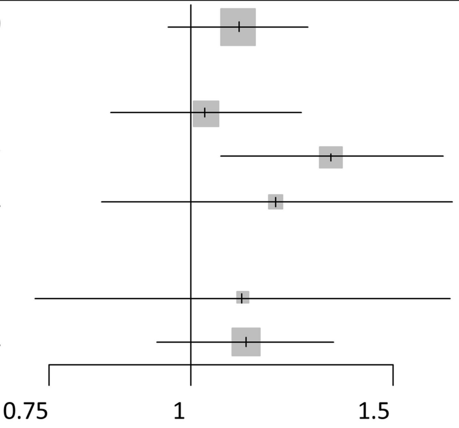

Supplement: S18 Fig — 95%CI: 95% Confidence Interval; P: P value. I2: between-strata heterogeneity. PHet: P value of between-strata heterogeneity. (PDF) [file pone.0177875.s018.pdf]
