# Supplementary material for: Obesity, metabolic factors and risk of different histological types of lung cancer: A Mendelian randomization study
Source: PLoS One. 2017 Jun 8;12(6):e0177875. doi: 10.1371/journal.pone.0177875 (PMC5464539; doi:10.1371/journal.pone.0177875)

S20 Fig - Forest plot of lung cancer risk for each SD increase in glucose 2h post-challenge observed in a likelihood-based MR approach. 95%CI: 95% Confidence Interval; P: P value. I^2: between-strata heterogeneity. PHet: P value of between-strata heterogeneity.

Lung Cancer	Cases	Controls	Odds Ratio	95% CI	P
Overall	29,266	56,450	0.96	0.77 - 1.20	0.75
Histology type				I^2 = 21%; **PHet**= 0.28	
Adenocarcinoma	11,273	55,483	1.10	0.82 - 1.49	0.52
Squamous cell	7,426	55,627	0.92	0.65 - 1.31	0.66
Small cell	2,664	21,444	0.66	0.38 - 1.16	0.15
Smoking group				I^2 = 0%; **PHet**= 0.32	
Never smokers	2,355	7,504	1.50	0.78 - 2.86	0.22
Ever smokers	23,223	16,964	1.05	0.80 - 1.38	0.74

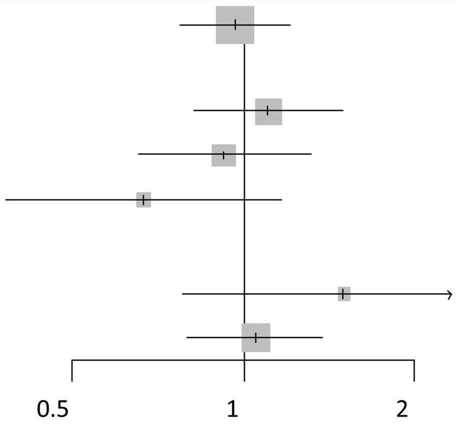

Supplement: S20 Fig — 95%CI: 95% Confidence Interval; P: P value. I2: between-strata heterogeneity. PHet: P value of between-strata heterogeneity. (PDF) [file pone.0177875.s020.pdf]
