# Supplementary material for: Obesity, metabolic factors and risk of different histological types of lung cancer: A Mendelian randomization study
Source: PLoS One. 2017 Jun 8;12(6):e0177875. doi: 10.1371/journal.pone.0177875 (PMC5464539; doi:10.1371/journal.pone.0177875)
Supplement: S1 Table — CHR: Chromosome. BP: Base pair. SE: Standard error. BMI: Body mass index. HDL: High-density lipoprotein, LDL: Low-density lipoprotein. (PDF) [file pone.0177875.s022.pdf]

S1 Table- Association parameters of instrumental SNPs for the corresponding metabolic factor and for different lung cancer groups. CHR: Chromosome. BP: Base pair. SE: Standard error. BMI: Body mass index. HDL: High-density lipoprotein, LDL: Low-density lipoprotein.

						SNP to phenotype effect		SNP to disease effect: Lung Overall		Adenocarcinoma		Squamous cell		Small cell		Overall ever smokers		Overall never smokers		
SNP	CHR	BP	Mean imputation quality	Effect allele	Other allele	Estimate	SE	Estiamte	SE	Estiamte	SE	Estiamte	SE	Estiamte	SE	Estiamte	SE	Estiamte	SE	Trait
rs657452	1	49589847	1.00	A	G	0.02	0.003	0.02	0.01	0.03	0.02	0.03	0.02	0.03	0.03	0.02	0.02	0.08	0.04	BMI
rs3101336	1	72751185	1.00	C	T	0.03	0.003	-0.01	0.01	-0.01	0.02	-0.01	0.02	-0.06	0.03	-0.02	0.02	0.03	0.04	BMI
rs12566985	1	75002193	1.00	G	A	0.02	0.003	0.00	0.01	0.00	0.02	-0.01	0.02	0.03	0.03	NA	NA	-0.06	0.04	BMI
rs12401738	1	78446761	1.00	A	G	0.02	0.003	0.05	0.01	0.06	0.02	0.05	0.02	0.03	0.03	0.06	0.02	0.01	0.04	BMI
rs11165643	1	96924097	1.00	T	C	0.02	0.003	0.03	0.01	0.02	0.02	0.00	0.02	0.04	0.03	NA	NA	0.05	0.04	BMI
rs17024393	1	110154688	1.00	C	T	0.07	0.009	-0.01	0.03	-0.03	0.05	0.06	0.05	0.03	0.08	-0.05	0.04	0.00	0.11	BMI
rs543874	1	177889480	1.00	G	A	0.05	0.004	0.00	0.02	0.01	0.02	0.01	0.02	0.02	0.04	0.02	0.02	0.02	0.05	BMI
rs2820292	1	201784287	1.00	C	A	0.02	0.003	0.03	0.01	0.03	0.02	0.02	0.02	0.03	0.03	0.02	0.01	0.00	0.04	BMI
rs10182181	2	25150296	1.00	G	A	0.03	0.003	0.00	0.01	0.00	0.02	0.02	0.02	-0.02	0.03	0.01	0.01	-0.02	0.04	BMI
rs11126666	2	26928811	1.00	A	G	0.02	0.003	-0.01	0.01	-0.04	0.02	0.00	0.02	-0.03	0.03	0.00	0.02	-0.03	0.04	BMI
rs1016287	2	59305625	1.00	T	C	0.02	0.003	-0.02	0.01	-0.02	0.02	-0.04	0.02	-0.04	0.03	-0.03	0.02	-0.01	0.04	BMI
rs11688816	2	63053048	1.00	G	A	0.02	0.003	-0.01	0.01	-0.02	0.02	-0.01	0.02	-0.04	0.03	0.00	0.01	-0.05	0.04	BMI
rs2121279	2	143043285	1.00	T	C	0.03	0.004	0.02	0.02	-0.02	0.02	0.04	0.03	0.02	0.04	0.01	0.02	0.02	0.05	BMI
rs1528435	2	181550962	0.75	T	C	0.02	0.003	0.00	0.01	-0.02	0.02	0.01	0.02	0.02	0.03	0.00	0.02	-0.01	0.04	BMI
rs7599312	2	213413231	1.00	G	A	0.02	0.003	-0.01	0.01	-0.01	0.02	0.01	0.02	0.03	0.03	0.02	0.02	-0.07	0.04	BMI
rs6804842	3	25106437	1.00	G	A	0.02	0.003	-0.01	0.01	0.00	0.02	-0.02	0.02	0.01	0.03	0.00	0.02	-0.02	0.04	BMI
rs2365389	3	61236462	0.99	C	T	0.02	0.003	0.00	0.01	0.00	0.02	0.00	0.02	0.00	0.03	-0.03	0.02	0.04	0.04	BMI
rs3849570	3	81792112	1.00	A	C	0.02	0.003	0.01	0.01	0.01	0.02	0.02	0.02	0.04	0.03	0.01	0.02	0.04	0.04	BMI
rs13078960	3	85807590	1.00	G	T	0.03	0.004	-0.02	0.01	-0.02	0.02	-0.03	0.02	0.00	0.04	0.00	0.02	-0.01	0.04	BMI
rs16851483	3	141275436	1.00	T	G	0.05	0.008	0.03	0.02	0.07	0.03	-0.02	0.04	0.06	0.06	0.06	0.03	0.02	0.07	BMI
rs1516725	3	185824004	0.97	C	T	0.05	0.005	-0.02	0.02	-0.03	0.02	-0.01	0.03	0.03	0.04	-0.02	0.02	0.01	0.05	BMI
rs10938397	4	45182527	0.98	G	A	0.04	0.003	-0.01	0.01	-0.01	0.02	-0.02	0.02	0.03	0.03	0.00	0.02	0.00	0.04	BMI
rs13107325	4	103188709	0.99	T	C	0.05	0.007	0.05	0.02	0.03	0.03	0.01	0.04	0.10	0.06	0.07	0.03	0.13	0.06	BMI
rs11727676	4	145659064	1.00	T	C	0.04	0.006	0.01	0.02	-0.01	0.03	0.02	0.03	-0.02	0.05	0.00	0.03	0.11	0.06	BMI
rs2112347	5	75015242	0.99	T	G	0.03	0.003	0.01	0.01	0.00	0.02	-0.01	0.02	0.02	0.03	0.01	0.02	-0.03	0.04	BMI
rs205262	6	34563164	1.00	G	A	0.02	0.004	0.01	0.01	0.01	0.02	0.01	0.02	0.03	0.03	0.02	0.02	-0.03	0.04	BMI
rs2033529	6	40348653	1.00	G	A	0.02	0.003	-0.02	0.01	-0.01	0.02	-0.03	0.02	-0.03	0.03	-0.03	0.02	-0.02	0.04	BMI
rs2207139	6	50845490	1.00	G	A	0.05	0.004	0.01	0.02	0.01	0.02	0.03	0.02	0.01	0.04	0.00	0.02	0.07	0.05	BMI
rs9400239	6	108977663	0.98	C	T	0.02	0.003	-0.02	0.01	-0.02	0.02	-0.02	0.02	-0.05	0.03	-0.03	0.02	-0.04	0.04	BMI
rs13191362	6	163033350	1.00	A	G	0.03	0.005	0.04	0.02	0.03	0.03	0.03	0.03	0.10	0.05	0.04	0.02	-0.01	0.06	BMI
rs1167827	7	75163169	1.00	G	A	0.02	0.003	0.02	0.01	0.03	0.02	0.01	0.02	0.00	0.03	0.03	0.01	0.00	0.04	BMI
rs2245368	7	76608143	1.00	C	T	0.03	0.006	-0.02	0.02	-0.01	0.02	-0.01	0.03	NA	NA	NA	NA	NA	NA	BMI
rs17405819	8	76806584	1.00	T	C	0.02	0.003	0.00	0.01	0.00	0.02	-0.01	0.02	0.04	0.03	0.01	0.02	-0.08	0.04	BMI
rs2033732	8	85079709	1.00	C	T	0.02	0.004	-0.01	0.01	0.00	0.02	-0.01	0.02	0.01	0.03	0.00	0.02	-0.04	0.04	BMI
rs4740619	9	15634326	0.99	T	C	0.02	0.003	0.00	0.01	0.00	0.02	0.01	0.02	0.01	0.03	0.01	0.01	-0.01	0.04	BMI
rs10968576	9	28414339	1.00	G	A	0.03	0.003	-0.01	0.01	-0.03	0.02	0.02	0.02	-0.05	0.03	0.00	0.02	-0.05	0.04	BMI
rs6477694	9	111932342	1.00	C	T	0.02	0.003	0.00	0.01	-0.01	0.02	0.02	0.02	0.03	0.03	0.01	0.02	-0.06	0.04	BMI
rs1928295	9	120378483	1.00	T	C	0.02	0.003	0.02	0.01	0.01	0.02	0.01	0.02	0.01	0.03	0.02	0.01	0.03	0.04	BMI
rs10733682	9	129460914	1.00	A	G	0.02	0.003	0.00	0.01	-0.02	0.02	0.01	0.02	0.01	0.03	0.01	0.01	0.00	0.03	BMI
rs7899106	10	87410904	1.00	G	A	0.04	0.007	-0.03	0.03	-0.04	0.04	0.07	0.04	0.05	0.07	-0.03	0.04	-0.07	0.08	BMI
rs17094222	10	102395440	1.00	C	T	0.03	0.004	-0.02	0.01	-0.01	0.02	-0.03	0.02	0.01	0.04	0.00	0.02	-0.03	0.04	BMI
rs11191560	10	104869038	1.00	C	T	0.03	0.005	0.03	0.02	0.02	0.03	0.06	0.03	0.03	0.05	0.02	0.03	0.01	0.06	BMI
rs7903146	10	114758349	1.00	C	T	0.02	0.003	0.01	0.01	0.02	0.02	0.01	0.02	0.07	0.03	0.03	0.02	-0.02	0.04	BMI
rs2316901	11	8679016	0.97	G	A	0.02	0.003	0.01	0.01	0.04	0.02	-0.03	0.02	-0.03	0.03	0.00	0.02	0.06	0.04	BMI
rs11030104	11	27684517	1.00	A	G	0.04	0.004	0.03	0.01	0.01	0.02	0.05	0.02	0.02	0.04	0.00	0.02	0.04	0.04	BMI
rs2176598	11	43864278	1.00	T	C	0.02	0.004	0.01	0.01	0.00	0.02	0.03	0.02	0.00	0.03	0.02	0.02	-0.06	0.04	BMI
rs3817334	11	47650993	0.99	T	C	0.03	0.003	-0.02	0.01	-0.04	0.02	0.00	0.02	-0.05	0.03	-0.01	0.02	-0.08	0.04	BMI
rs12286929	11	115022404	1.00	G	A	0.02	0.003	0.01	0.01	0.01	0.02	0.02	0.02	-0.02	0.03	0.03	0.01	-0.01	0.04	BMI
rs7138803	12	50247468	1.00	A	G	0.03	0.003	-0.03	0.01	0.01	0.02	-0.08	0.02	-0.06	0.03	-0.05	0.02	0.00	0.04	BMI

rs11057405	12	122781897	1.00	G	A	0.03	0.006	-0.01	0.02	0.02	0.03	-0.04	0.03	0.05	0.05	-0.02	0.03	0.06	0.06	BMI
rs9581854	13	28017782	0.99	T	C	0.03	0.005	-0.01	0.02	-0.05	0.02	0.00	0.02	0.00	0.04	0.01	0.02	-0.07	0.05	BMI
rs12429545	13	54102206	1.00	A	G	0.03	0.005	0.01	0.02	0.01	0.02	0.02	0.03	0.03	0.04	0.03	0.02	-0.03	0.05	BMI
rs10132280	14	25928179	0.96	C	A	0.02	0.003	0.01	0.01	-0.03	0.02	0.02	0.02	0.03	0.03	-0.01	0.02	-0.02	0.04	BMI
rs12885454	14	29736838	0.98	C	A	0.02	0.003	0.02	0.01	0.00	0.02	0.03	0.02	0.09	0.03	0.03	0.02	0.10	0.04	BMI
rs11847697	14	30515112	1.00	T	C	0.05	0.008	0.02	0.03	-0.03	0.04	0.00	0.05	0.18	0.07	0.03	0.04	-0.03	0.09	BMI
rs7141420	14	79899454	1.00	T	C	0.02	0.003	0.00	0.01	-0.01	0.02	0.00	0.02	0.03	0.03	0.00	0.01	-0.01	0.03	BMI
rs3736485	15	51748610	0.97	A	G	0.02	0.003	0.00	0.01	0.01	0.02	0.01	0.02	-0.02	0.03	0.01	0.02	0.04	0.04	BMI
rs16951275	15	68077168	1.00	T	C	0.03	0.004	0.04	0.01	0.03	0.02	0.05	0.02	0.03	0.04	0.03	0.02	0.02	0.04	BMI
rs758747	16	3627358	0.82	T	C	0.02	0.004	0.02	0.01	0.02	0.02	0.01	0.02	0.03	0.03	0.02	0.02	0.05	0.04	BMI
rs12446632	16	19935389	1.00	G	A	0.04	0.005	-0.01	0.02	-0.02	0.02	0.02	0.03	0.00	0.04	-0.02	0.02	0.00	0.05	BMI
rs3888190	16	28889486	0.95	A	C	0.03	0.003	0.03	0.01	0.01	0.02	0.04	0.02	0.05	0.03	0.04	0.02	-0.06	0.04	BMI
rs9925964	16	31129895	1.00	A	G	0.02	0.003	0.02	0.01	0.01	0.02	0.03	0.02	0.04	0.03	0.01	0.02	0.08	0.04	BMI
rs1421085	16	53800954	1.00	C	T	0.08	0.003	-0.01	0.01	-0.03	0.02	0.00	0.02	0.04	0.03	-0.02	0.02	-0.05	0.04	BMI
rs1000940	17	5283252	1.00	G	A	0.02	0.003	0.02	0.01	0.01	0.02	0.03	0.02	0.05	0.03	0.01	0.02	0.05	0.04	BMI
rs12940622	17	78615571	1.00	G	A	0.02	0.003	0.01	0.01	0.00	0.02	0.01	0.02	0.07	0.03	0.01	0.01	0.01	0.04	BMI
rs1808579	18	21104888	1.00	C	T	0.02	0.003	0.02	0.01	0.02	0.02	0.00	0.02	0.05	0.03	0.04	0.01	0.04	0.04	BMI
rs7243357	18	56883319	1.00	T	G	0.02	0.004	-0.02	0.02	0.00	0.02	-0.02	0.02	0.00	0.04	-0.02	0.02	-0.03	0.05	BMI
rs6567160	18	57829135	0.99	C	T	0.06	0.004	0.00	0.01	-0.01	0.02	0.01	0.02	-0.03	0.03	0.00	0.02	-0.09	0.04	BMI
rs17724992	19	18454825	1.00	A	G	0.02	0.004	0.01	0.01	0.01	0.02	0.02	0.02	0.05	0.03	0.00	0.02	0.00	0.04	BMI
rs29941	19	34309532	0.99	G	A	0.02	0.003	-0.01	0.01	0.00	0.02	0.01	0.02	-0.02	0.03	0.00	0.02	-0.04	0.04	BMI
rs2075650	19	45395619	1.00	A	G	0.03	0.005	0.05	0.02	0.02	0.02	0.07	0.03	0.14	0.04	0.04	0.02	0.05	0.05	BMI
rs2287019	19	46202172	1.00	C	T	0.04	0.004	-0.02	0.01	-0.03	0.02	0.01	0.02	0.01	0.04	-0.01	0.02	-0.03	0.04	BMI
rs3810291	19	47569003	1.00	A	G	0.03	0.004	0.01	0.01	0.01	0.02	-0.02	0.02	-0.02	0.03	0.00	0.02	0.05	0.04	BMI
rs2765539	1	119549418	0.99	T	C	0.03	0.004	-0.01	0.01	0.01	0.02	-0.04	0.02	-0.03	0.03	-0.02	0.02	0.02	0.04	Waist-to-hip ratio
rs1011731	1	172346548	0.99	G	A	0.02	0.003	0.00	0.01	0.00	0.02	-0.03	0.02	0.05	0.03	0.01	0.01	-0.05	0.04	Waist-to-hip ratio
rs1563355	1	219653101	1.00	C	T	0.03	0.004	0.01	0.01	0.04	0.02	-0.01	0.02	0.01	0.03	0.03	0.02	-0.04	0.04	Waist-to-hip ratio
rs929641	2	58792377	1.00	A	G	0.02	0.003	0.03	0.01	0.00	0.02	0.04	0.02	0.01	0.03	0.04	0.01	-0.02	0.04	Waist-to-hip ratio
rs1128249	2	165528624	0.99	G	T	0.02	0.003	0.02	0.01	0.02	0.02	0.00	0.02	0.03	0.03	0.03	0.02	0.04	0.04	Waist-to-hip ratio
rs1569135	2	188115398	1.00	A	G	0.02	0.003	-0.01	0.01	-0.03	0.02	0.01	0.02	0.01	0.03	-0.01	0.01	-0.01	0.03	Waist-to-hip ratio
rs2972164	3	12334416	0.99	C	T	0.02	0.003	-0.01	0.01	-0.02	0.02	0.00	0.02	0.01	0.03	-0.01	0.01	-0.05	0.04	Waist-to-hip ratio
rs9860730	3	64701146	0.98	A	G	0.02	0.004	0.01	0.01	0.00	0.02	0.04	0.02	0.02	0.03	0.01	0.02	0.03	0.04	Waist-to-hip ratio
rs17451107	3	156797609	1.00	T	C	0.02	0.004	-0.01	0.01	-0.01	0.02	0.02	0.02	-0.03	0.03	0.00	0.02	-0.05	0.04	Waist-to-hip ratio
rs459193	5	55806751	1.00	A	G	0.03	0.004	-0.01	0.01	-0.02	0.02	-0.01	0.02	-0.04	0.03	-0.02	0.02	0.06	0.04	Waist-to-hip ratio
rs1294421	6	6743149	1.00	G	T	0.03	0.003	0.02	0.01	0.00	0.02	0.05	0.02	0.05	0.03	0.01	0.02	-0.03	0.04	Waist-to-hip ratio
rs11755724	6	7118990	0.96	G	A	0.02	0.004	-0.02	0.01	0.00	0.02	-0.02	0.02	-0.05	0.03	-0.02	0.02	-0.07	0.04	Waist-to-hip ratio
rs998584	6	43757896	1.00	A	C	0.03	0.004	0.00	0.01	-0.01	0.02	0.01	0.02	0.03	0.03	0.04	0.02	0.04	0.04	Waist-to-hip ratio
rs2745359	6	127381956	0.83	C	T	0.06	0.009	0.00	0.03	-0.01	0.04	0.06	0.05	-0.14	0.07	-0.04	0.04	-0.04	0.09	Waist-to-hip ratio
rs10245353	7	25858614	0.99	A	C	0.03	0.004	0.02	0.02	0.00	0.02	0.06	0.02	0.03	0.04	0.01	0.02	0.01	0.05	Waist-to-hip ratio
rs7801581	7	27223771	1.00	T	C	0.02	0.004	-0.02	0.01	-0.01	0.02	-0.02	0.02	0.00	0.03	-0.02	0.02	-0.02	0.04	Waist-to-hip ratio
rs12549058	8	72492238	0.99	G	T	0.04	0.006	0.03	0.02	0.03	0.03	0.04	0.04	0.10	0.06	0.02	0.03	-0.01	0.07	Waist-to-hip ratio
rs4929927	11	8658485	0.98	G	A	0.02	0.003	0.01	0.01	0.04	0.02	-0.03	0.02	-0.03	0.03	0.00	0.02	0.06	0.04	Waist-to-hip ratio
rs11048470	12	26487283	1.00	T	G	0.03	0.004	0.00	0.01	0.02	0.02	-0.03	0.02	-0.05	0.03	0.00	0.02	0.00	0.04	Waist-to-hip ratio
rs10783615	12	54349773	0.86	G	A	0.04	0.005	0.02	0.02	0.04	0.02	-0.01	0.03	-0.01	0.04	0.02	0.02	0.10	0.05	Waist-to-hip ratio
rs10876528	12	54421476	1.00	A	C	0.03	0.004	0.02	0.01	0.02	0.02	0.02	0.02	0.03	0.03	0.02	0.02	0.01	0.04	Waist-to-hip ratio
rs1316952	12	124399550	1.00	T	C	0.03	0.005	0.00	0.02	-0.03	0.02	-0.02	0.03	-0.01	0.04	-0.01	0.02	-0.03	0.05	Waist-to-hip ratio
rs1440372	15	67033151	0.97	C	T	0.02	0.004	-0.02	0.01	0.00	0.02	-0.04	0.02	0.01	0.03	-0.03	0.02	-0.04	0.04	Waist-to-hip ratio
rs4640244	17	21284223	1.00	G	A	0.02	0.004	0.00	0.01	-0.01	0.02	0.01	0.02	-0.01	0.03	-0.01	0.02	0.01	0.04	Waist-to-hip ratio
rs3786897	19	33893008	1.00	G	A	0.02	0.003	-0.03	0.01	-0.03	0.02	-0.05	0.02	-0.03	0.03	-0.02	0.02	-0.01	0.04	Waist-to-hip ratio
rs2075650	19	45395619	1.00	A	G	0.03	0.005	0.05	0.02	0.02	0.02	0.07	0.03	0.14	0.04	0.04	0.02	0.05	0.05	Waist-to-hip ratio
rs16996700	20	50981945	1.00	T	C	0.02	0.004	0.01	0.01	0.01	0.02	0.00	0.02	0.03	0.03	0.01	0.02	0.00	0.04	Waist-to-hip ratio
rs2179129	22	29450923	0.99	A	G	0.02	0.003	0.01	0.01	0.02	0.02	-0.02	0.02	0.01	0.03	0.00	0.01	0.04	0.04	Waist-to-hip ratio
rs12748152	1	27138393	0.98	C	T	0.05	0.006	0.02	0.02	0.03	0.03	0.04	0.03	0.03	0.05	0.02	0.03	0.11	0.07	HDL
rs4660293	1	40028180	0.99	A	G	0.04	0.004	0.01	0.01	0.01	0.02	-0.01	0.02	-0.01	0.03	0.00	0.02	0.07	0.04	HDL
rs12145743	1	156700651	0.99	G	T	0.02	0.004	-0.02	0.01	0.00	0.02	-0.02	0.02	0.00	0.03	-0.04	0.02	0.09	0.04	HDL
rs4650994	1	178515312	1.00	G	A	0.02	0.003	0.01	0.01	0.01	0.02	0.02	0.02	0.03	0.03	0.01	0.01	0.03	0.04	HDL
rs1689800	1	182168885	0.98	A	G	0.03	0.004	0.02	0.01	0.01	0.02	0.00	0.02	0.04	0.03	0.02	0.02	0.05	0.04	HDL

rs4846914	1	230295691	1.00	A	G	0.05	0.003	0.00	0.01	0.00	0.02	-0.02	0.02	-0.02	0.03	-0.01	0.02	0.10	0.04	HDL
rs1042034	2	21225281	1.00	C	T	0.07	0.004	-0.01	0.01	-0.02	0.02	-0.02	0.02	-0.02	0.03	-0.02	0.02	-0.02	0.04	HDL
rs12328675	2	165540800	0.98	C	T	0.04	0.005	-0.01	0.02	0.01	0.02	-0.03	0.03	-0.03	0.04	-0.01	0.02	-0.06	0.05	HDL
rs1047891	2	211540507	1.00	C	A	0.03	0.004	0.00	0.01	-0.02	0.02	0.01	0.02	0.05	0.03	-0.01	0.02	0.01	0.04	HDL
rs2972146	2	227100698	1.00	G	T	0.03	0.004	-0.01	0.01	-0.02	0.02	-0.03	0.02	-0.04	0.03	-0.03	0.02	0.01	0.04	HDL
rs2606736	3	11400249	1.00	C	T	0.02	0.004	-0.01	0.01	-0.02	0.02	0.01	0.02	-0.04	0.03	-0.02	0.02	0.00	0.04	HDL
rs2290547	3	47061183	0.93	G	A	0.03	0.005	-0.01	0.02	-0.02	0.02	-0.03	0.02	-0.01	0.04	-0.03	0.02	0.05	0.05	HDL
rs2013208	3	50129399	1.00	T	C	0.03	0.004	-0.02	0.01	-0.01	0.02	-0.02	0.02	-0.06	0.03	-0.01	0.01	-0.03	0.04	HDL
rs13326165	3	52532118	1.00	A	G	0.03	0.004	-0.02	0.01	-0.04	0.02	-0.02	0.02	0.00	0.04	-0.01	0.02	-0.08	0.04	HDL
rs6805251	3	119560606	1.00	T	C	0.02	0.004	-0.02	0.01	0.00	0.02	-0.04	0.02	-0.03	0.03	-0.02	0.02	0.02	0.04	HDL
rs3822072	4	89741269	0.99	G	A	0.03	0.003	-0.01	0.01	0.01	0.02	-0.01	0.02	0.00	0.03	-0.01	0.01	0.08	0.04	HDL
rs2602836	4	100014805	1.00	A	G	0.02	0.003	0.00	0.01	-0.03	0.02	0.04	0.02	-0.01	0.03	0.01	0.01	-0.06	0.04	HDL
rs13107325	4	103188709	0.99	C	T	0.07	0.008	-0.05	0.02	-0.03	0.03	-0.01	0.04	-0.10	0.06	-0.07	0.03	-0.13	0.06	HDL
rs6450176	5	53298025	1.00	G	A	0.03	0.004	0.01	0.01	0.01	0.02	0.02	0.02	0.05	0.03	0.02	0.02	0.05	0.04	HDL
rs2814944	6	34552797	1.00	G	A	0.03	0.005	-0.01	0.02	-0.02	0.02	0.01	0.03	-0.03	0.04	-0.01	0.02	-0.01	0.05	HDL
rs998584	6	43757896	1.00	C	A	0.03	0.004	0.00	0.01	0.01	0.02	-0.01	0.02	-0.03	0.03	-0.04	0.02	-0.04	0.04	HDL
rs1936800	6	127436064	1.00	C	T	0.02	0.003	-0.01	0.01	-0.02	0.02	-0.02	0.02	-0.01	0.03	-0.01	0.01	-0.01	0.04	HDL
rs702485	7	6449272	0.99	G	A	0.02	0.003	-0.01	0.01	-0.02	0.02	-0.01	0.02	0.02	0.03	-0.01	0.01	-0.07	0.04	HDL
rs4142995	7	17919258	0.99	G	T	0.03	0.004	0.00	0.01	0.00	0.02	0.01	0.02	0.02	0.03	0.02	0.02	-0.04	0.04	HDL
rs4917014	7	50305863	1.00	G	T	0.02	0.004	0.00	0.01	-0.03	0.02	0.03	0.02	-0.01	0.03	0.01	0.02	-0.08	0.04	HDL
rs17145738	7	72982874	1.00	T	C	0.04	0.005	0.04	0.02	0.01	0.03	0.09	0.03	0.15	0.04	0.04	0.02	0.02	0.06	HDL
rs4731702	7	130433384	0.98	T	C	0.03	0.003	0.00	0.01	0.01	0.02	0.00	0.02	-0.01	0.03	0.01	0.01	-0.04	0.04	HDL
rs17173637	7	150529449	1.00	T	C	0.04	0.006	-0.03	0.02	-0.04	0.03	-0.06	0.03	-0.10	0.05	-0.04	0.03	-0.04	0.06	HDL
rs9987289	8	9183358	0.99	G	A	0.08	0.006	-0.04	0.02	-0.03	0.03	-0.06	0.03	0.00	0.05	-0.03	0.03	-0.01	0.06	HDL
rs12678919	8	19844222	1.00	G	A	0.16	0.006	-0.01	0.02	-0.03	0.03	-0.03	0.03	0.00	0.05	0.00	0.02	-0.08	0.06	HDL
rs2293889	8	116599199	0.99	G	T	0.03	0.004	0.00	0.01	-0.01	0.02	-0.02	0.02	-0.02	0.03	-0.02	0.02	-0.04	0.04	HDL
rs638491	9	15290012	0.99	G	A	0.04	0.005	0.00	0.02	-0.04	0.02	0.03	0.03	-0.05	0.04	-0.01	0.02	-0.03	0.05	HDL
rs1883025	9	107664301	1.00	C	T	0.07	0.004	-0.03	0.01	-0.01	0.02	0.00	0.02	-0.07	0.03	-0.01	0.02	-0.06	0.04	HDL
rs970548	10	46013277	1.00	C	A	0.03	0.004	0.00	0.01	0.00	0.02	0.02	0.02	0.01	0.03	0.00	0.02	-0.03	0.04	HDL
rs2923084	11	10388782	1.00	A	G	0.03	0.005	0.01	0.02	-0.01	0.02	0.03	0.02	0.03	0.04	0.01	0.02	0.03	0.04	HDL
rs11246602	11	51512090	0.86	C	T	0.03	0.005	0.03	0.02	0.01	0.03	0.01	0.03	-0.01	0.05	NA	NA	-0.03	0.06	HDL
rs174546	11	61569830	1.00	C	T	0.04	0.004	0.03	0.01	0.03	0.02	0.03	0.02	0.02	0.03	0.05	0.02	0.02	0.04	HDL
rs12801636	11	65391317	1.00	A	G	0.02	0.004	-0.01	0.01	-0.02	0.02	-0.01	0.02	0.02	0.03	-0.01	0.02	-0.02	0.04	HDL
rs499974	11	75455021	1.00	C	A	0.03	0.004	-0.01	0.02	-0.03	0.02	0.03	0.02	-0.01	0.04	0.01	0.02	-0.07	0.05	HDL
rs3741298	11	116657561	0.97	T	C	0.05	0.006	-0.01	0.01	0.00	0.02	-0.03	0.02	0.06	0.04	0.01	0.02	-0.03	0.04	HDL
rs7134375	12	20473758	0.96	A	C	0.02	0.004	0.01	0.01	0.03	0.02	0.00	0.02	-0.02	0.03	0.01	0.02	-0.01	0.04	HDL
rs11613352	12	57792580	1.00	T	C	0.03	0.004	0.02	0.01	0.02	0.02	0.01	0.02	0.04	0.03	0.01	0.02	0.00	0.04	HDL
rs7134594	12	110000193	1.00	T	C	0.04	0.005	0.01	0.01	0.00	0.02	0.01	0.02	-0.02	0.03	0.00	0.01	0.00	0.04	HDL
rs4759375	12	123796238	1.00	T	C	0.06	0.010	0.01	0.02	-0.01	0.03	0.03	0.03	0.10	0.05	0.02	0.03	-0.01	0.06	HDL
rs4765127	12	124460167	1.00	T	G	0.03	0.005	-0.02	0.01	-0.02	0.02	0.00	0.02	-0.06	0.03	-0.02	0.02	0.01	0.04	HDL
rs838880	12	125261593	0.98	C	T	0.05	0.004	-0.02	0.01	0.01	0.02	-0.02	0.02	0.04	0.03	-0.03	0.02	-0.04	0.04	HDL
rs4983559	14	105277209	1.00	G	A	0.02	0.004	0.01	0.01	0.00	0.02	0.02	0.02	0.02	0.03	0.01	0.02	0.03	0.04	HDL
rs1532085	15	58683366	1.00	A	G	0.11	0.004	0.01	0.01	0.01	0.02	0.01	0.02	0.04	0.03	0.01	0.02	-0.01	0.04	HDL
rs1121980	16	53809247	1.00	G	A	0.02	0.003	0.01	0.01	0.03	0.02	-0.01	0.02	-0.02	0.03	0.01	0.01	0.04	0.04	HDL
rs3764261	16	56993324	1.00	A	C	0.24	0.004	0.00	0.01	0.00	0.02	0.01	0.02	0.00	0.03	0.00	0.02	0.03	0.04	HDL
rs16942887	16	67928042	1.00	A	G	0.08	0.005	-0.03	0.02	-0.04	0.02	0.02	0.03	0.00	0.04	-0.03	0.02	0.01	0.05	HDL
rs1877031	17	37814080	1.00	A	G	0.03	0.004	0.01	0.01	0.02	0.02	0.00	0.02	0.02	0.03	0.00	0.02	0.06	0.04	HDL
rs4148005	17	66882466	0.99	T	G	0.03	0.004	-0.01	0.01	0.01	0.02	-0.04	0.02	0.00	0.03	0.01	0.02	-0.04	0.04	HDL
rs4129767	17	76403984	0.94	A	G	0.02	0.003	0.01	0.01	0.01	0.02	-0.01	0.02	0.06	0.03	0.02	0.01	-0.03	0.04	HDL
rs7241918	18	47160953	1.00	T	G	0.09	0.006	0.02	0.02	0.05	0.02	0.00	0.02	0.02	0.04	0.05	0.02	-0.06	0.05	HDL
rs12967135	18	57849023	1.00	G	A	0.03	0.005	0.00	0.01	0.01	0.02	-0.01	0.02	0.03	0.03	0.00	0.02	0.09	0.04	HDL
rs7255436	19	8433196	0.93	A	C	0.03	0.005	0.01	0.01	0.02	0.02	0.01	0.02	0.06	0.03	0.00	0.01	0.07	0.04	HDL
rs737337	19	11347493	1.00	T	C	0.06	0.006	0.04	0.02	0.03	0.03	0.04	0.03	0.02	0.05	0.06	0.03	-0.02	0.06	HDL
rs731839	19	33899065	1.00	A	G	0.02	0.004	-0.03	0.01	-0.01	0.02	-0.05	0.02	-0.05	0.03	-0.02	0.02	-0.02	0.04	HDL
rs4420638	19	45422946	1.00	A	G	0.07	0.007	0.06	0.02	0.05	0.02	0.10	0.03	0.07	0.04	0.06	0.02	0.03	0.05	HDL
rs17695224	19	52324216	1.00	G	A	0.03	0.004	0.00	0.01	0.01	0.02	0.00	0.02	0.04	0.03	0.02	0.02	0.01	0.04	HDL
rs103294	19	54797848	1.00	T	C	0.05	0.004	0.02	0.02	0.02	0.02	0.03	0.02	0.01	0.04	0.02	0.02	0.07	0.05	HDL

rs6065906	20	44554015	1.00	T	C	0.06	0.004	0.01	0.01	-0.01	0.02	0.05	0.02	-0.01	0.04	0.01	0.02	0.10	0.05	HDL
rs1801177	8	19805708	0.99	G	A	0.20	0.031	-0.04	0.05	-0.10	0.07	0.00	0.08	-0.14	0.13	0.01	0.06	NA	NA	HDL rare
rs268	8	19813529	1.00	A	G	0.23	0.031	0.00	0.05	-0.07	0.06	0.15	0.08	-0.04	0.11	0.01	0.06	-0.12	0.14	HDL rare
rs186808413	11	117042408	0.86	T	C	0.29	0.035	0.02	0.08	0.08	0.10	0.14	0.12	NA	NA	NA	NA	NA	NA	HDL rare
rs1800777	16	57017319	0.78	G	A	0.40	0.018	0.05	0.03	0.04	0.05	0.13	0.06	-0.05	0.09	-0.02	0.04	0.03	0.11	HDL rare
rs1672867	16	57071114	0.73	G	A	0.17	0.020	-0.02	0.05	-0.01	0.07	-0.07	0.08	NA	NA	NA	NA	NA	NA	HDL rare
rs72836561	17	41926126	1.00	C	T	0.20	0.019	0.02	0.04	0.02	0.06	-0.02	0.07	NA	NA	NA	NA	NA	NA	HDL rare
rs77960347	18	47109955	0.93	G	A	0.29	0.031	0.02	0.05	0.03	0.07	0.14	0.09	-0.13	0.14	0.03	0.07	NA	NA	HDL rare
rs1800961	20	43042364	0.99	C	T	0.15	0.016	0.06	0.03	0.04	0.05	0.04	0.05	0.10	0.08	0.04	0.04	0.02	0.11	HDL rare
rs10903129	1	25768937	1.00	G	A	0.03	0.004	0.00	0.01	-0.01	0.02	0.02	0.02	-0.04	0.03	0.01	0.01	-0.05	0.03	LDL
rs12748152	1	27138393	0.98	T	C	0.05	0.007	-0.02	0.02	-0.03	0.03	-0.04	0.03	-0.03	0.05	-0.02	0.03	-0.11	0.07	LDL
rs2479409	1	55504650	1.00	G	A	0.06	0.004	0.00	0.01	-0.01	0.02	0.01	0.02	0.05	0.03	0.01	0.02	0.00	0.04	LDL
rs2131925	1	63025942	1.00	T	G	0.05	0.004	-0.01	0.01	-0.02	0.02	-0.03	0.02	0.05	0.03	-0.01	0.02	-0.01	0.04	LDL
rs267733	1	150958836	1.00	A	G	0.03	0.005	-0.03	0.02	-0.03	0.02	-0.02	0.03	-0.03	0.04	-0.01	0.02	-0.09	0.05	LDL
rs2642442	1	220973563	0.97	T	C	0.04	0.005	-0.01	0.01	-0.02	0.02	-0.01	0.02	0.04	0.03	0.00	0.02	-0.08	0.04	LDL
rs484084	1	234857676	0.97	C	T	0.03	0.005	0.00	0.01	0.02	0.02	-0.02	0.02	0.00	0.03	0.00	0.01	0.01	0.04	LDL
rs1367117	2	21263900	1.00	A	G	0.12	0.004	0.01	0.01	0.01	0.02	0.05	0.02	0.01	0.03	0.01	0.02	-0.01	0.04	LDL
rs4299376	2	44072576	1.00	G	T	0.08	0.005	-0.02	0.01	-0.01	0.02	-0.04	0.02	-0.02	0.03	-0.02	0.02	-0.02	0.04	LDL
rs2710642	2	63149557	1.00	A	G	0.02	0.004	0.00	0.01	-0.02	0.02	0.02	0.02	-0.02	0.03	0.00	0.02	-0.01	0.04	LDL
rs10490626	2	118835841	1.00	G	A	0.05	0.007	0.02	0.02	0.00	0.03	0.04	0.03	0.11	0.05	0.03	0.03	-0.01	0.06	LDL
rs2030746	2	121309488	1.00	T	C	0.02	0.004	0.00	0.01	0.03	0.02	-0.01	0.02	-0.04	0.03	-0.01	0.01	0.05	0.04	LDL
rs1250229	2	216304384	0.98	C	T	0.02	0.004	0.01	0.01	-0.01	0.02	0.06	0.02	0.01	0.03	0.02	0.02	-0.02	0.04	LDL
rs11563251	2	234679384	1.00	T	C	0.03	0.006	-0.02	0.02	0.01	0.03	-0.06	0.03	0.02	0.05	-0.03	0.02	-0.02	0.06	LDL
rs7640978	3	32533010	0.97	C	T	0.04	0.007	0.00	0.02	-0.02	0.03	0.01	0.03	0.01	0.05	-0.01	0.03	-0.09	0.06	LDL
rs17404153	3	132163200	0.97	G	T	0.03	0.005	-0.02	0.02	-0.07	0.02	0.01	0.03	0.00	0.04	-0.02	0.02	-0.06	0.05	LDL
rs6831256	4	3473139	0.98	G	A	0.02	0.004	0.00	0.01	0.01	0.02	-0.03	0.02	0.02	0.03	0.01	0.01	-0.01	0.04	LDL
rs4530754	5	122855416	1.00	A	G	0.03	0.004	0.00	0.01	0.01	0.02	0.02	0.02	-0.05	0.03	0.01	0.01	0.00	0.04	LDL
rs6882076	5	156390297	1.00	C	T	0.05	0.004	-0.01	0.01	0.01	0.02	-0.02	0.02	-0.04	0.03	-0.02	0.02	0.06	0.04	LDL
rs3757354	6	16127407	0.98	C	T	0.04	0.004	-0.02	0.01	-0.02	0.02	0.00	0.02	-0.05	0.03	-0.03	0.02	-0.09	0.04	LDL
rs1800562	6	26093141	1.00	G	A	0.06	0.008	-0.03	0.03	-0.07	0.04	0.02	0.04	0.08	0.07	-0.04	0.03	-0.13	0.08	LDL
rs3798236	6	116309649	0.99	T	C	0.02	0.004	-0.02	0.01	0.00	0.02	-0.01	0.02	-0.04	0.03	-0.02	0.02	0.00	0.04	LDL
rs12670798	7	21607352	0.99	C	T	0.03	0.004	0.03	0.01	0.01	0.02	0.04	0.02	0.02	0.03	0.03	0.02	0.02	0.04	LDL
rs4722551	7	25991826	0.90	C	T	0.04	0.005	0.00	0.02	0.00	0.02	0.01	0.03	-0.07	0.04	-0.01	0.02	0.09	0.05	LDL
rs9987289	8	9183358	0.99	G	A	0.07	0.007	-0.04	0.02	-0.03	0.03	-0.06	0.03	0.00	0.05	-0.03	0.03	-0.01	0.06	LDL
rs10102164	8	55421614	1.00	A	G	0.03	0.005	0.00	0.01	0.00	0.02	-0.02	0.02	0.01	0.04	-0.02	0.02	0.05	0.05	LDL
rs10808546	8	126495818	1.00	C	T	0.05	0.004	-0.03	0.01	-0.03	0.02	-0.01	0.02	-0.03	0.03	-0.03	0.01	-0.02	0.04	LDL
rs11136341	8	145043543	1.00	G	A	0.04	0.006	0.01	0.01	-0.01	0.02	0.03	0.02	0.02	0.03	0.01	0.02	0.03	0.04	LDL
rs3780181	9	2640759	1.00	A	G	0.04	0.007	-0.01	0.02	-0.01	0.03	-0.01	0.04	0.02	0.06	-0.04	0.03	-0.03	0.07	LDL
rs2255141	10	113933886	0.96	A	G	0.03	0.004	0.00	0.01	0.00	0.02	-0.01	0.02	0.01	0.03	0.00	0.02	-0.04	0.04	LDL
rs11220462	11	126243952	0.99	A	G	0.06	0.006	0.00	0.02	-0.01	0.02	-0.01	0.03	0.02	0.04	-0.02	0.02	0.07	0.05	LDL
rs11065987	12	112072424	1.00	A	G	0.03	0.004	0.04	0.01	0.02	0.02	0.04	0.02	0.06	0.03	0.03	0.02	0.03	0.04	LDL
rs1169288	12	121416650	0.97	C	A	0.04	0.004	0.01	0.01	0.02	0.02	-0.03	0.02	-0.01	0.03	0.00	0.02	0.00	0.04	LDL
rs4942486	13	32953388	1.00	T	C	0.02	0.004	0.04	0.01	0.05	0.02	0.04	0.02	0.07	0.03	0.04	0.01	0.09	0.03	LDL
rs8017377	14	24883887	1.00	A	G	0.03	0.004	0.01	0.01	0.02	0.02	0.02	0.02	0.03	0.03	0.02	0.01	0.02	0.04	LDL
rs3764261	16	56993324	1.00	C	A	0.05	0.004	0.00	0.01	0.00	0.02	-0.01	0.02	0.00	0.03	0.00	0.02	-0.03	0.04	LDL
rs2000999	16	72108093	0.99	A	G	0.07	0.005	0.01	0.01	0.00	0.02	-0.01	0.02	0.08	0.04	0.00	0.02	0.01	0.04	LDL
rs7206971	17	45425115	1.00	A	G	0.03	0.006	0.00	0.01	-0.01	0.02	0.01	0.02	0.02	0.03	0.01	0.01	-0.01	0.04	LDL
rs1801689	17	64210580	0.99	C	A	0.10	0.014	-0.04	0.04	-0.03	0.05	0.05	0.06	-0.14	0.09	NA	NA	-0.32	0.09	LDL
rs6511720	19	11202306	1.00	G	T	0.22	0.006	-0.03	0.02	0.00	0.03	-0.06	0.03	-0.05	0.05	-0.04	0.02	-0.01	0.05	LDL
rs10401969	19	19407718	1.00	T	C	0.12	0.007	0.01	0.02	0.04	0.03	-0.01	0.04	0.06	0.06	0.03	0.03	-0.04	0.07	LDL
rs4420638	19	45422946	1.00	G	A	0.23	0.008	-0.06	0.02	-0.05	0.02	-0.10	0.03	-0.07	0.04	-0.06	0.02	-0.03	0.05	LDL
rs364585	20	12962718	0.99	G	A	0.02	0.004	-0.02	0.01	-0.02	0.02	-0.01	0.02	-0.03	0.03	-0.02	0.02	-0.04	0.04	LDL
rs2328223	20	17845921	1.00	C	A	0.03	0.005	-0.02	0.01	-0.03	0.02	-0.01	0.02	-0.10	0.04	-0.01	0.02	-0.03	0.04	LDL
rs2902940	20	39091487	1.00	A	G	0.03	0.004	0.01	0.01	-0.02	0.02	0.02	0.02	0.11	0.03	0.00	0.02	0.01	0.04	LDL
rs6016505	20	39678289	0.97	T	C	0.04	0.005	0.00	0.01	0.01	0.02	0.02	0.02	-0.03	0.03	0.00	0.01	0.02	0.04	LDL
rs5763662	22	30378703	0.99	T	C	0.08	0.012	0.02	0.04	0.03	0.05	0.02	0.06	0.08	0.10	-0.01	0.05	0.07	0.11	LDL
rs4253772	22	46627603	0.97	T	C	0.03	0.006	0.00	0.02	0.00	0.03	-0.01	0.03	0.07	0.05	-0.02	0.02	0.13	0.06	LDL

rs1077514	1	23766233	1.00	T	C	0.03	0.005	0.00	0.02	-0.02	0.02	-0.01	0.03	0.03	0.04	0.01	0.02	-0.01	0.05	Total cholesterol
rs10903129	1	25768937	1.00	G	A	0.03	0.004	0.00	0.01	-0.01	0.02	0.02	0.02	-0.04	0.03	0.01	0.01	-0.05	0.03	Total cholesterol
rs2479409	1	55504650	1.00	G	A	0.05	0.004	0.00	0.01	-0.01	0.02	0.01	0.02	0.05	0.03	0.01	0.02	0.00	0.04	Total cholesterol
rs2131925	1	63025942	1.00	T	G	0.07	0.004	-0.01	0.01	-0.02	0.02	-0.03	0.02	0.05	0.03	-0.01	0.02	-0.01	0.04	Total cholesterol
rs7515577	1	93009438	0.99	A	C	0.04	0.006	-0.03	0.01	-0.01	0.02	-0.02	0.02	-0.07	0.04	-0.01	0.02	-0.07	0.04	Total cholesterol
rs2642442	1	220973563	0.97	T	C	0.04	0.005	-0.01	0.01	-0.02	0.02	-0.01	0.02	0.04	0.03	0.00	0.02	-0.08	0.04	Total cholesterol
rs484084	1	234857676	0.97	C	T	0.03	0.005	0.00	0.01	0.02	0.02	-0.02	0.02	0.00	0.03	0.00	0.01	0.01	0.04	Total cholesterol
rs1367117	2	21263900	1.00	A	G	0.10	0.004	0.01	0.01	0.01	0.02	0.05	0.02	0.01	0.03	0.01	0.02	-0.01	0.04	Total cholesterol
rs1260326	2	27730940	0.98	T	C	0.05	0.004	0.00	0.01	-0.01	0.02	0.00	0.02	0.00	0.03	-0.02	0.02	-0.04	0.04	Total cholesterol
rs4299376	2	44072576	1.00	G	T	0.08	0.004	-0.02	0.01	-0.01	0.02	-0.04	0.02	-0.02	0.03	-0.02	0.02	-0.02	0.04	Total cholesterol
rs10490626	2	118835841	1.00	G	A	0.04	0.007	0.02	0.02	0.00	0.03	0.04	0.03	0.11	0.05	0.03	0.03	-0.01	0.06	Total cholesterol
rs2030746	2	121309488	1.00	T	C	0.02	0.004	0.00	0.01	0.03	0.02	-0.01	0.02	-0.04	0.03	-0.01	0.01	0.05	0.04	Total cholesterol
rs7570971	2	135837906	0.81	A	C	0.03	0.004	-0.02	0.01	-0.02	0.02	-0.04	0.02	0.00	0.03	-0.03	0.02	-0.04	0.04	Total cholesterol
rs2287623	2	169830155	0.99	G	A	0.03	0.004	0.00	0.01	0.00	0.02	0.02	0.02	-0.02	0.03	0.00	0.02	0.01	0.04	Total cholesterol
rs11694172	2	203532304	1.00	G	A	0.03	0.004	0.01	0.01	0.01	0.02	-0.01	0.02	0.01	0.03	0.02	0.02	-0.05	0.04	Total cholesterol
rs11563251	2	234679384	1.00	T	C	0.04	0.006	-0.02	0.02	0.01	0.03	-0.06	0.03	0.02	0.05	-0.03	0.02	-0.02	0.06	Total cholesterol
rs7956	3	12624763	0.98	T	C	0.03	0.006	0.00	0.01	0.00	0.02	0.02	0.02	0.01	0.04	0.03	0.02	-0.06	0.04	Total cholesterol
rs7640978	3	32533010	0.97	C	T	0.04	0.007	0.00	0.02	-0.02	0.03	0.01	0.03	0.01	0.05	-0.01	0.03	-0.09	0.06	Total cholesterol
rs13315871	3	58381287	0.99	G	A	0.04	0.006	0.05	0.02	0.05	0.03	0.08	0.03	-0.05	0.05	0.02	0.03	0.08	0.06	Total cholesterol
rs6831256	4	3473139	0.98	G	A	0.02	0.004	0.00	0.01	0.01	0.02	-0.03	0.02	0.02	0.03	0.01	0.01	-0.01	0.04	Total cholesterol
rs4530754	5	122855416	1.00	A	G	0.02	0.004	0.00	0.01	0.01	0.02	0.02	0.02	-0.05	0.03	0.01	0.01	0.00	0.04	Total cholesterol
rs6882076	5	156390297	1.00	C	T	0.05	0.004	-0.01	0.01	0.01	0.02	-0.02	0.02	-0.04	0.03	-0.02	0.02	0.06	0.04	Total cholesterol
rs3757354	6	16127407	0.98	C	T	0.03	0.004	-0.02	0.01	-0.02	0.02	0.00	0.02	-0.05	0.03	-0.03	0.02	-0.09	0.04	Total cholesterol
rs1800562	6	26093141	1.00	G	A	0.06	0.008	-0.03	0.03	-0.07	0.04	0.02	0.04	0.08	0.07	-0.04	0.03	-0.13	0.08	Total cholesterol
rs2814982	6	34546560	0.99	C	T	0.04	0.006	0.00	0.02	0.02	0.03	-0.01	0.03	-0.08	0.05	0.00	0.02	0.02	0.06	Total cholesterol
rs2758886	6	39250837	0.99	A	G	0.02	0.004	0.01	0.01	0.01	0.02	0.01	0.02	-0.01	0.03	0.00	0.02	0.02	0.04	Total cholesterol
rs3798236	6	116309649	0.99	T	C	0.03	0.004	-0.02	0.01	0.00	0.02	-0.01	0.02	-0.04	0.03	-0.02	0.02	0.00	0.04	Total cholesterol
rs9376090	6	135411228	0.98	T	C	0.03	0.004	-0.02	0.01	0.00	0.02	-0.01	0.02	-0.08	0.03	-0.02	0.02	0.04	0.04	Total cholesterol
rs1997243	7	1083777	0.99	G	A	0.03	0.005	0.00	0.02	0.01	0.02	-0.03	0.03	-0.03	0.04	-0.01	0.02	-0.01	0.05	Total cholesterol
rs12670798	7	21607352	0.99	C	T	0.04	0.004	0.03	0.01	0.01	0.02	0.04	0.02	0.02	0.03	0.03	0.02	0.02	0.04	Total cholesterol
rs4722551	7	25991826	0.90	C	T	0.03	0.005	0.00	0.02	0.00	0.02	0.01	0.03	-0.07	0.04	-0.01	0.02	0.09	0.05	Total cholesterol
rs9987289	8	9183358	0.99	G	A	0.08	0.006	-0.04	0.02	-0.03	0.03	-0.06	0.03	0.00	0.05	-0.03	0.03	-0.01	0.06	Total cholesterol
rs1495741	8	18272881	1.00	G	A	0.03	0.006	0.00	0.01	0.00	0.02	0.01	0.02	0.01	0.03	0.00	0.02	-0.01	0.04	Total cholesterol
rs10102164	8	55421614	1.00	A	G	0.03	0.004	0.00	0.01	0.00	0.02	-0.02	0.02	0.01	0.04	-0.02	0.02	0.05	0.05	Total cholesterol
rs2737229	8	116648565	0.97	A	C	0.03	0.004	-0.02	0.01	-0.01	0.02	-0.03	0.02	-0.03	0.03	-0.01	0.02	-0.01	0.04	Total cholesterol
rs10808546	8	126495818	1.00	C	T	0.06	0.004	-0.03	0.01	-0.03	0.02	-0.01	0.02	-0.03	0.03	-0.03	0.01	-0.02	0.04	Total cholesterol
rs11136341	8	145043543	1.00	G	A	0.04	0.006	0.01	0.01	-0.01	0.02	0.03	0.02	0.02	0.03	0.01	0.02	0.03	0.04	Total cholesterol
rs3780181	9	2640759	1.00	A	G	0.04	0.007	-0.01	0.02	-0.01	0.03	-0.01	0.04	0.02	0.06	-0.04	0.03	-0.03	0.07	Total cholesterol
rs638491	9	15290012	0.99	G	A	0.03	0.005	0.00	0.02	-0.04	0.02	0.03	0.03	-0.05	0.04	-0.01	0.02	-0.03	0.05	Total cholesterol
rs1883025	9	107664301	1.00	C	T	0.07	0.004	-0.03	0.01	-0.01	0.02	0.00	0.02	-0.07	0.03	-0.01	0.02	-0.06	0.04	Total cholesterol
rs10904908	10	17260290	1.00	G	A	0.03	0.004	0.02	0.01	0.04	0.02	0.01	0.02	0.00	0.03	0.02	0.02	0.06	0.04	Total cholesterol
rs970548	10	46013277	1.00	C	A	0.03	0.004	0.00	0.01	0.00	0.02	0.02	0.02	0.01	0.03	0.00	0.02	-0.03	0.04	Total cholesterol
rs2255141	10	113933886	0.96	A	G	0.03	0.004	0.00	0.01	0.00	0.02	-0.01	0.02	0.01	0.03	0.00	0.02	-0.04	0.04	Total cholesterol
rs10128711	11	18632984	1.00	C	T	0.03	0.004	0.02	0.01	0.03	0.02	0.00	0.02	0.00	0.03	0.01	0.02	0.07	0.04	Total cholesterol
rs174546	11	61569830	1.00	C	T	0.05	0.004	0.03	0.01	0.03	0.02	0.03	0.02	0.02	0.03	0.05	0.02	0.02	0.04	Total cholesterol
rs3741298	11	116657561	0.97	C	T	0.07	0.006	0.01	0.01	0.00	0.02	0.03	0.02	-0.06	0.04	-0.01	0.02	0.03	0.04	Total cholesterol
rs11603023	11	118486067	1.00	T	C	0.02	0.004	-0.03	0.01	-0.03	0.02	-0.04	0.02	0.00	0.03	-0.03	0.01	-0.03	0.04	Total cholesterol
rs11220462	11	126243952	0.99	A	G	0.05	0.006	0.00	0.02	-0.01	0.02	-0.01	0.03	0.02	0.04	-0.02	0.02	0.07	0.05	Total cholesterol
rs11065987	12	112072424	1.00	A	G	0.03	0.004	0.04	0.01	0.02	0.02	0.04	0.02	0.06	0.03	0.03	0.02	0.03	0.04	Total cholesterol
rs1169288	12	121416650	0.97	C	A	0.03	0.004	0.01	0.01	0.02	0.02	-0.03	0.02	-0.01	0.03	0.00	0.02	0.00	0.04	Total cholesterol
rs1532085	15	58683366	1.00	A	G	0.05	0.004	0.01	0.01	0.01	0.02	0.01	0.02	0.04	0.03	0.01	0.02	-0.01	0.04	Total cholesterol
rs3764261	16	56993324	1.00	A	C	0.05	0.004	0.00	0.01	0.00	0.02	0.01	0.02	0.00	0.03	0.00	0.02	0.03	0.04	Total cholesterol
rs2000999	16	72108093	0.99	A	G	0.06	0.004	0.01	0.01	0.00	0.02	-0.01	0.02	0.08	0.04	0.00	0.02	0.01	0.04	Total cholesterol
rs7206971	17	45425115	1.00	A	G	0.03	0.005	0.00	0.01	-0.01	0.02	0.01	0.02	0.02	0.03	0.01	0.01	-0.01	0.04	Total cholesterol
rs7241918	18	47160953	1.00	T	G	0.06	0.007	0.02	0.02	0.05	0.02	0.00	0.02	0.02	0.04	0.05	0.02	-0.06	0.05	Total cholesterol
rs6511720	19	11202306	1.00	G	T	0.19	0.006	-0.03	0.02	0.00	0.03	-0.06	0.03	-0.05	0.05	-0.04	0.02	-0.01	0.05	Total cholesterol
rs10401969	19	19407718	1.00	T	C	0.14	0.007	0.01	0.02	0.04	0.03	-0.01	0.04	0.06	0.06	0.03	0.03	-0.04	0.07	Total cholesterol

rs4420638	19	45422946	1.00	G	A	0.20	0.007	-0.06	0.02	-0.05	0.02	-0.10	0.03	-0.07	0.04	-0.06	0.02	-0.03	0.05	Total cholesterol
rs492602	19	49206417	1.00	G	A	0.03	0.004	0.03	0.01	0.08	0.02	-0.03	0.02	0.03	0.03	0.01	0.01	0.11	0.04	Total cholesterol
rs2277862	20	34152782	0.99	C	T	0.03	0.005	-0.01	0.02	-0.01	0.02	-0.02	0.03	-0.01	0.04	-0.01	0.02	0.03	0.05	Total cholesterol
rs2902940	20	39091487	1.00	A	G	0.02	0.004	0.01	0.01	-0.02	0.02	0.02	0.02	0.11	0.03	0.00	0.02	0.01	0.04	Total cholesterol
rs6016505	20	39678289	0.97	T	C	0.04	0.005	0.00	0.01	0.01	0.02	0.02	0.02	-0.03	0.03	0.00	0.01	0.02	0.04	Total cholesterol
rs1800961	20	43042364	0.99	C	T	0.11	0.010	0.06	0.03	0.04	0.05	0.04	0.05	0.10	0.08	0.04	0.04	0.02	0.11	Total cholesterol
rs138777	22	35711098	0.97	A	G	0.02	0.004	-0.02	0.01	-0.01	0.02	-0.03	0.02	0.00	0.03	-0.03	0.02	0.01	0.04	Total cholesterol
rs4253772	22	46627603	0.97	T	C	0.03	0.006	0.00	0.02	0.00	0.03	-0.01	0.03	0.07	0.05	-0.02	0.02	0.13	0.06	Total cholesterol
rs11591147	1	55505647	0.99	G	T	0.49	0.029	0.01	0.05	-0.02	0.07	0.02	0.08	NA	NA	NA	NA	NA	NA	non-HDL rare
rs533617	2	21233972	1.00	T	C	0.18	0.015	0.05	0.03	0.04	0.04	0.06	0.05	0.03	0.07	0.07	0.04	0.10	0.10	non-HDL rare
rs12691202	2	21249716	1.00	C	T	0.14	0.018	-0.05	0.03	-0.06	0.05	-0.04	0.05	-0.01	0.08	-0.08	0.04	-0.07	0.10	non-HDL rare
rs28477226	19	43965804	0.80	G	T	0.28	0.048	0.07	0.15	0.04	0.20	NA	NA	NA	NA	NA	NA	NA	NA	non-HDL rare
rs1058402	19	45150614	0.99	G	A	0.10	0.015	0.01	0.03	0.01	0.04	-0.01	0.04	0.00	0.07	0.03	0.04	0.03	0.08	non-HDL rare
rs3208856	19	45296806	0.99	C	T	0.15	0.019	0.03	0.04	0.07	0.05	0.04	0.06	-0.13	0.09	0.03	0.05	NA	NA	non-HDL rare
rs12748152	1	27138393	0.98	T	C	0.04	0.006	-0.02	0.02	-0.03	0.03	-0.04	0.03	-0.03	0.05	-0.02	0.03	-0.11	0.07	Triglycerides
rs2131925	1	63025942	1.00	T	G	0.07	0.004	-0.01	0.01	-0.02	0.02	-0.03	0.02	0.05	0.03	-0.01	0.02	-0.01	0.04	Triglycerides
rs4846914	1	230295691	1.00	G	A	0.04	0.003	0.00	0.01	0.00	0.02	0.02	0.02	0.02	0.03	0.01	0.02	-0.10	0.04	Triglycerides
rs1042034	2	21225281	1.00	T	C	0.07	0.004	0.01	0.01	0.02	0.02	0.02	0.02	0.02	0.03	0.02	0.02	0.02	0.04	Triglycerides
rs1260326	2	27730940	0.98	T	C	0.11	0.003	0.00	0.01	-0.01	0.02	0.00	0.02	0.00	0.03	-0.02	0.02	-0.04	0.04	Triglycerides
rs10195252	2	165513091	1.00	T	C	0.03	0.004	0.01	0.01	0.01	0.02	0.00	0.02	0.03	0.03	0.02	0.02	0.04	0.04	Triglycerides
rs2972146	2	227100698	1.00	T	G	0.03	0.003	0.01	0.01	0.02	0.02	0.03	0.02	0.04	0.03	0.03	0.02	-0.01	0.04	Triglycerides
rs645040	3	135926622	0.99	T	G	0.03	0.004	0.00	0.01	0.01	0.02	0.02	0.02	0.02	0.04	0.01	0.02	-0.08	0.04	Triglycerides
rs6831256	4	3473139	0.98	G	A	0.03	0.004	0.00	0.01	0.01	0.02	-0.03	0.02	0.02	0.03	0.01	0.01	-0.01	0.04	Triglycerides
rs442177	4	88030261	0.99	T	G	0.03	0.003	0.02	0.01	0.01	0.02	0.03	0.02	0.01	0.03	0.02	0.02	0.02	0.04	Triglycerides
rs9686661	5	55861786	1.00	T	C	0.04	0.004	0.02	0.02	0.02	0.02	0.00	0.02	0.06	0.04	0.03	0.02	0.03	0.04	Triglycerides
rs6882076	5	156390297	1.00	C	T	0.03	0.004	-0.01	0.01	0.01	0.02	-0.02	0.02	-0.04	0.03	-0.02	0.02	0.06	0.04	Triglycerides
rs998584	6	43757896	1.00	A	C	0.03	0.004	0.00	0.01	-0.01	0.02	0.01	0.02	0.03	0.03	0.04	0.02	0.04	0.04	Triglycerides
rs1936800	6	127436064	1.00	T	C	0.02	0.003	0.01	0.01	0.02	0.02	0.02	0.02	0.01	0.03	0.01	0.01	0.01	0.04	Triglycerides
rs4722551	7	25991826	0.90	T	C	0.03	0.004	0.00	0.02	0.00	0.02	-0.01	0.03	0.07	0.04	0.01	0.02	-0.09	0.05	Triglycerides
rs17145738	7	72982874	1.00	C	T	0.11	0.005	-0.04	0.02	-0.01	0.03	-0.09	0.03	-0.15	0.04	-0.04	0.02	-0.02	0.06	Triglycerides
rs38855	7	116358044	1.00	A	G	0.02	0.003	-0.01	0.01	-0.01	0.02	0.03	0.02	-0.03	0.03	-0.01	0.01	0.01	0.04	Triglycerides
rs2271357	8	10683623	1.00	A	G	0.02	0.004	0.01	0.01	0.03	0.02	0.00	0.02	0.02	0.03	0.01	0.02	0.05	0.04	Triglycerides
rs1495741	8	18272881	1.00	G	A	0.04	0.006	0.00	0.01	0.00	0.02	0.01	0.02	0.01	0.03	0.00	0.02	-0.01	0.04	Triglycerides
rs12678919	8	19844222	1.00	A	G	0.17	0.006	0.01	0.02	0.03	0.03	0.03	0.03	0.00	0.05	0.00	0.02	0.08	0.06	Triglycerides
rs10808546	8	126495818	1.00	C	T	0.08	0.003	-0.03	0.01	-0.03	0.02	-0.01	0.02	-0.03	0.03	-0.03	0.01	-0.02	0.04	Triglycerides
rs7080386	10	65048306	0.99	C	A	0.03	0.005	0.02	0.01	0.01	0.02	0.03	0.02	0.04	0.03	0.01	0.01	0.03	0.04	Triglycerides
rs2068888	10	94839642	1.00	G	A	0.02	0.003	0.00	0.01	0.01	0.02	0.01	0.02	-0.01	0.03	0.00	0.01	0.07	0.04	Triglycerides
rs174546	11	61569830	1.00	T	C	0.04	0.003	-0.03	0.01	-0.03	0.02	-0.03	0.02	-0.02	0.03	-0.05	0.02	-0.02	0.04	Triglycerides
rs3741298	11	116657561	0.97	C	T	0.15	0.006	0.01	0.01	0.00	0.02	0.03	0.02	-0.06	0.04	-0.01	0.02	0.03	0.04	Triglycerides
rs11613352	12	57792580	1.00	C	T	0.03	0.004	-0.02	0.01	-0.02	0.02	-0.01	0.02	-0.04	0.03	-0.01	0.02	0.00	0.04	Triglycerides
rs4765127	12	124460167	1.00	G	T	0.03	0.005	0.02	0.01	0.02	0.02	0.00	0.02	0.06	0.03	0.02	0.02	-0.01	0.04	Triglycerides
rs2412710	15	42683787	1.00	A	G	0.10	0.013	-0.09	0.04	-0.08	0.05	-0.13	0.07	NA	NA	-0.17	0.05	-0.07	0.12	Triglycerides
rs1532085	15	58683366	1.00	A	G	0.03	0.003	0.01	0.01	0.01	0.02	0.01	0.02	0.04	0.03	0.01	0.02	-0.01	0.04	Triglycerides
rs3198697	16	15129940	0.95	C	T	0.02	0.003	-0.02	0.01	-0.02	0.02	-0.03	0.02	-0.03	0.03	-0.03	0.02	0.03	0.04	Triglycerides
rs1121980	16	53809247	1.00	A	G	0.02	0.003	-0.01	0.01	-0.03	0.02	0.01	0.02	0.02	0.03	-0.01	0.01	-0.04	0.04	Triglycerides
rs8077889	17	41878166	0.97	C	A	0.03	0.004	0.01	0.01	0.00	0.02	0.02	0.02	0.03	0.04	0.01	0.02	0.03	0.04	Triglycerides
rs7248104	19	7224431	1.00	G	A	0.02	0.003	0.00	0.01	0.00	0.02	-0.02	0.02	0.04	0.03	0.00	0.01	0.03	0.04	Triglycerides
rs10401969	19	19407718	1.00	T	C	0.12	0.007	0.01	0.02	0.04	0.03	-0.01	0.04	0.06	0.06	0.03	0.03	-0.04	0.07	Triglycerides
rs731839	19	33899065	1.00	G	A	0.02	0.004	0.03	0.01	0.01	0.02	0.05	0.02	0.05	0.03	0.02	0.02	0.02	0.04	Triglycerides
rs439401	19	45414451	1.00	C	T	0.07	0.004	0.01	0.01	0.02	0.02	0.00	0.02	-0.02	0.03	0.01	0.02	0.05	0.04	Triglycerides
rs6065906	20	44554015	1.00	C	T	0.05	0.004	-0.01	0.01	0.01	0.02	-0.05	0.02	0.01	0.04	-0.01	0.02	-0.10	0.05	Triglycerides
rs5756931	22	38546033	0.99	T	C	0.02	0.004	0.02	0.01	-0.02	0.02	0.06	0.02	0.03	0.03	0.02	0.02	-0.01	0.04	Triglycerides
rs184070214	6	31526080	1.00	A	G	0.11	0.016	-0.10	0.04	-0.08	0.06	-0.15	0.07	-0.06	0.11	NA	NA	NA	NA	Triglycerides rare
rs12721041	11	116693871	0.79	T	C	0.20	0.025	0.01	0.05	0.07	0.07	0.04	0.08	-0.15	0.13	-0.01	0.07	0.13	0.16	Triglycerides rare
rs186808413	11	117042408	0.86	C	T	0.27	0.033	-0.02	0.08	-0.08	0.10	-0.14	0.12	NA	NA	NA	NA	NA	NA	Triglycerides rare
rs143659874	11	117222691	0.76	A	C	0.21	0.039	0.11	0.11	0.13	0.14	0.10	0.18	NA	NA	0.35	0.15	NA	NA	Triglycerides rare
rs55830029	15	42179479	0.89	T	C	0.20	0.036	-0.04	0.12	-0.21	0.17	0.09	0.20	NA	NA	NA	NA	NA	NA	Triglycerides rare

rs55707100	15	43820717	1.00	T	C	0.13	0.020	-0.09	0.03	-0.03	0.04	-0.18	0.05	-0.09	0.08	-0.13	0.04	-0.08	0.10	Triglycerides rare
rs116843064	19	8429323	0.99	G	A	0.24	0.021	0.02	0.04	-0.05	0.06	0.01	0.07	NA	NA	NA	NA	NA	NA	Triglycerides rare
rs340874	1	214159256	0.99	C	T	0.02	0.003	-0.02	0.01	-0.04	0.02	-0.01	0.02	-0.09	0.03	-0.03	0.01	-0.02	0.04	Fasting glucose
rs780094	2	27741237	1.00	C	T	0.04	0.003	0.00	0.01	0.01	0.02	0.00	0.02	0.02	0.03	0.01	0.02	0.04	0.04	Fasting glucose
rs560887	2	169763148	1.00	C	T	0.09	0.003	0.00	0.01	0.01	0.02	0.03	0.02	-0.02	0.03	0.01	0.02	0.00	0.04	Fasting glucose
rs11715915	3	49455330	1.00	C	T	0.02	0.003	0.03	0.01	0.01	0.02	0.04	0.02	0.06	0.03	0.01	0.02	0.02	0.04	Fasting glucose
rs11708067	3	123065778	1.00	A	G	0.03	0.003	-0.03	0.01	-0.04	0.02	0.00	0.02	-0.01	0.04	-0.01	0.02	-0.06	0.04	Fasting glucose
rs7651090	3	185513392	1.00	G	A	0.02	0.003	-0.02	0.01	-0.02	0.02	-0.02	0.02	0.01	0.03	-0.02	0.02	0.00	0.04	Fasting glucose
rs4869272	5	95539448	1.00	T	C	0.02	0.003	-0.01	0.01	0.00	0.02	0.00	0.02	0.00	0.03	-0.01	0.02	0.00	0.04	Fasting glucose
rs9368222	6	20686996	1.00	A	C	0.02	0.003	-0.01	0.01	0.00	0.02	0.02	0.02	-0.10	0.03	0.00	0.02	-0.01	0.04	Fasting glucose
rs2191349	7	15064309	1.00	T	G	0.04	0.003	0.00	0.01	-0.02	0.02	0.00	0.02	0.07	0.03	0.00	0.01	-0.04	0.04	Fasting glucose
rs2908289	7	44223942	0.95	A	G	0.08	0.004	0.03	0.02	0.03	0.02	0.03	0.03	0.08	0.04	0.03	0.02	0.04	0.05	Fasting glucose
rs6943153	7	50791579	1.00	T	C	0.02	0.003	-0.02	0.01	-0.03	0.02	0.00	0.02	0.03	0.03	-0.01	0.02	-0.05	0.04	Fasting glucose
rs11558471	8	118185733	0.99	A	G	0.04	0.003	0.01	0.01	0.00	0.02	0.01	0.02	0.03	0.03	-0.02	0.02	0.03	0.04	Fasting glucose
rs10814916	9	4293150	0.95	C	A	0.02	0.003	0.01	0.01	0.00	0.02	-0.01	0.02	0.05	0.03	0.00	0.01	0.03	0.04	Fasting glucose
rs10811661	9	22134094	1.00	T	C	0.03	0.004	0.00	0.02	-0.03	0.02	0.01	0.02	-0.06	0.04	-0.01	0.02	-0.05	0.05	Fasting glucose
rs3829109	9	139256766	1.00	G	A	0.02	0.004	0.00	0.01	-0.02	0.02	-0.02	0.02	-0.04	0.03	0.00	0.02	-0.04	0.04	Fasting glucose
rs7901695	10	114754088	0.99	C	T	0.03	0.003	-0.02	0.01	-0.02	0.02	-0.02	0.02	-0.09	0.03	-0.03	0.02	0.02	0.04	Fasting glucose
rs11605924	11	45873091	1.00	A	C	0.03	0.003	0.01	0.01	0.01	0.02	0.01	0.02	0.07	0.03	0.01	0.01	0.05	0.04	Fasting glucose
rs174576	11	61603510	1.00	C	A	0.03	0.003	0.03	0.01	0.03	0.02	0.03	0.02	0.03	0.03	0.05	0.02	0.02	0.04	Fasting glucose
rs11020124	11	92690661	0.98	C	T	0.08	0.003	0.01	0.01	0.00	0.02	0.01	0.02	0.01	0.03	0.01	0.02	0.01	0.04	Fasting glucose
rs10747083	12	133041618	1.00	A	G	0.02	0.003	0.01	0.01	0.02	0.02	0.06	0.02	-0.04	0.03	0.00	0.02	0.00	0.04	Fasting glucose
rs576674	13	33554302	0.93	G	A	0.02	0.004	-0.03	0.02	-0.01	0.02	-0.01	0.03	-0.04	0.04	-0.02	0.02	-0.01	0.05	Fasting glucose
rs3783347	14	100839261	0.99	G	T	0.02	0.003	0.03	0.01	0.03	0.02	0.04	0.02	0.00	0.04	0.03	0.02	-0.01	0.04	Fasting glucose
rs4502156	15	62383155	0.96	T	C	0.03	0.003	0.00	0.01	0.01	0.02	0.01	0.02	-0.03	0.03	0.02	0.02	0.03	0.04	Fasting glucose
rs6113722	20	22557099	0.99	G	A	0.05	0.007	0.00	0.03	0.01	0.04	0.03	0.05	0.02	0.07	0.01	0.04	0.00	0.08	Fasting glucose
rs6072275	20	39743905	1.00	A	G	0.02	0.004	0.00	0.02	0.00	0.02	0.03	0.02	-0.07	0.04	0.00	0.02	0.02	0.05	Fasting glucose
rs2820436	1	219640680	1.00	C	A	0.03	0.005	-0.01	0.01	-0.03	0.02	0.01	0.02	-0.03	0.03	-0.03	0.02	0.02	0.04	Fasting insulin
rs1530559	2	135755629	0.89	A	G	0.03	0.005	0.03	0.01	0.03	0.02	0.06	0.02	-0.02	0.03	0.03	0.02	0.05	0.04	Fasting insulin
rs10195252	2	165513091	1.00	T	C	0.03	0.005	0.01	0.01	0.01	0.02	0.00	0.02	0.03	0.03	0.02	0.02	0.04	0.04	Fasting insulin
rs974801	4	106071064	1.00	G	A	0.03	0.005	0.04	0.01	0.02	0.02	0.03	0.02	0.07	0.03	0.04	0.02	-0.01	0.04	Fasting insulin
rs4865796	5	53272664	1.00	A	G	0.03	0.005	-0.02	0.01	-0.03	0.02	-0.03	0.02	-0.01	0.03	-0.03	0.02	-0.02	0.04	Fasting insulin
rs459193	5	55806751	1.00	G	A	0.03	0.005	0.01	0.01	0.02	0.02	0.01	0.02	0.04	0.03	0.02	0.02	-0.06	0.04	Fasting insulin
rs2745353	6	127452935	1.00	T	C	0.03	0.004	0.00	0.01	0.02	0.02	0.02	0.02	0.02	0.03	0.02	0.01	0.00	0.04	Fasting insulin
rs1167800	7	75176196	0.98	A	G	0.03	0.005	0.01	0.01	0.03	0.02	-0.01	0.02	-0.02	0.03	0.01	0.01	-0.02	0.04	Fasting insulin
rs983309	8	9177732	0.99	T	G	0.05	0.007	0.04	0.02	0.02	0.03	0.07	0.03	-0.03	0.05	0.02	0.02	0.03	0.06	Fasting insulin
rs7903146	10	114758349	1.00	C	T	0.03	0.005	0.01	0.01	0.02	0.02	0.01	0.02	0.07	0.03	0.03	0.02	-0.02	0.04	Fasting insulin
rs731839	19	33899065	1.00	G	A	0.03	0.005	0.03	0.01	0.01	0.02	0.05	0.02	0.05	0.03	0.02	0.02	0.02	0.04	Fasting insulin
rs2877716	3	123094451	1.00	C	T	0.05	0.008	-0.03	0.01	-0.03	0.02	0.01	0.02	-0.05	0.03	0.00	0.02	-0.05	0.04	2-hour post-challenge glucose
rs1019503	5	96254817	1.00	A	G	0.04	0.006	0.00	0.01	0.01	0.02	-0.01	0.02	0.01	0.03	0.01	0.01	0.05	0.04	2-hour post-challenge glucose
rs6975024	7	44231886	0.99	C	T	0.06	0.009	0.04	0.02	0.02	0.02	0.04	0.03	0.08	0.04	0.03	0.02	0.04	0.05	2-hour post-challenge glucose
rs11782386	8	9201787	1.00	C	T	0.06	0.010	-0.03	0.02	-0.02	0.03	-0.03	0.03	0.00	0.05	0.01	0.02	-0.01	0.06	2-hour post-challenge glucose
rs12255372	10	114808902	1.00	T	G	0.05	0.008	-0.01	0.01	-0.01	0.02	-0.01	0.02	-0.08	0.03	-0.02	0.02	0.03	0.04	2-hour post-challenge glucose
rs11672660	19	46180184	0.97	T	C	0.07	0.008	0.02	0.01	0.04	0.02	0.00	0.02	-0.01	0.04	0.02	0.02	0.05	0.04	2-hour post-challenge glucose
