# Supplementary material for: Obesity, metabolic factors and risk of different histological types of lung cancer: A Mendelian randomization study
Source: PLoS One. 2017 Jun 8;12(6):e0177875. doi: 10.1371/journal.pone.0177875 (PMC5464539; doi:10.1371/journal.pone.0177875)
Supplement: S2 Table — HDL: High-density lipoprotein, LDL: Low-density lipoprotein. Chol: Cholesterol. OR: Odds ratio. LCI: Lower confidence interval. UCI: Upper confidence interval. P: P value. (PDF) [file pone.0177875.s023.pdf]

S2 Table- Risk incrase on lung cancer phenotypes for each standard deviation increase in the phenotype provided by weighted median MR approach. HDL: High-density lipoprotein, LDL: Low-density lipoprotein. Chol: Cholesterol. OR: Odds ratio. LCI: Lower confidence interval. UCI: Upper confidence interval. P: P value.

		**Lung Overall**					**Adenocarcinoma**					**Squamous cell**					**Small cell**					**Overall never smokers**					**Overall ever smokers**				
**Risk factor**	**N SNPs**	**OR**	**LCI**	**UCI**	**P**	**P Het**	**OR**	**LCI**	**UCI**	**P**	**P Het**	**OR**	**LCI**	**UCI**	**P**	**P Het**	**OR**	**LCI**	**UCI**	**P**	**P Het**	**OR**	**LCI**	**UCI**	**P**	**P Het**	**OR**	**LCI**	**UCI**	**P**	**P Het**
Body mass index	72	0.97	0.81	1.16	0.77	7.5E-05	0.88	0.70	1.11	0.29	0.07	1.15	0.85	1.56	0.36	0.01	1.26	0.77	2.05	0.36	0.09	0.68	0.40	1.14	0.14	0.16	1.07	0.86	1.33	0.55	0.01
Waist-hip ratio	31	1.00	0.76	1.31	0.97	0.05	0.88	0.61	1.29	0.54	0.16	1.26	0.80	2.00	0.32	0.02	1.01	0.51	2.00	0.98	0.32	0.65	0.30	1.40	0.28	0.55	0.89	0.63	1.25	0.50	0.16
HDL	62	1.02	0.93	1.12	0.64	0.01	1.00	0.87	1.14	0.99	0.43	1.05	0.90	1.21	0.56	4.7E-03	1.05	0.83	1.32	0.71	0.02	1.12	0.84	1.50	0.45	0.01	1.04	0.92	1.18	0.54	0.02
HDL rare variants	8	1.07	0.93	1.24	0.34	0.51	1.06	0.87	1.30	0.58	0.69	1.41	1.11	1.79	0.01	0.21	0.75	0.52	1.08	0.12	0.70	0.91	0.58	1.41	0.67	0.99	0.90	0.75	1.09	0.30	0.82
LDL	48	0.91	0.81	1.02	0.10	0.11	0.88	0.76	1.02	0.10	0.14	0.77	0.63	0.94	0.01	0.06	0.87	0.65	1.17	0.37	4.0E-03	0.90	0.67	1.20	0.48	0.04	0.89	0.77	1.02	0.10	0.34
Total Chol	65	0.90	0.81	1.01	0.09	0.01	0.99	0.86	1.14	0.88	0.03	0.80	0.66	0.97	0.02	0.03	0.86	0.65	1.14	0.31	0.01	0.88	0.66	1.18	0.40	0.44	0.88	0.76	1.02	0.08	0.19
Non-HDL rare variants	6	1.10	0.92	1.31	0.31	0.36	1.04	0.82	1.32	0.78	0.58	1.15	0.86	1.53	0.36	0.62	1.11	0.72	1.71	0.64	0.23	1.38	0.80	2.38	0.25	0.78	1.02	0.80	1.29	0.89	0.11
Triglycerides	38	1.01	0.90	1.14	0.86	0.01	1.05	0.89	1.23	0.60	0.46	1.14	0.94	1.37	0.18	8.9E-04	0.81	0.59	1.10	0.18	0.02	0.98	0.70	1.36	0.90	0.22	0.93	0.80	1.09	0.37	4.6E-03
Triglycerides rare variants	7	1.12	0.84	1.49	0.45	0.05	0.85	0.58	1.26	0.43	0.42	0.84	0.52	1.34	0.47	0.08	1.44	0.71	2.90	0.31	0.42	1.41	0.63	3.13	0.41	0.36	1.06	0.70	1.60	0.79	9.0E-04
Fasting glucose	24	1.16	0.95	1.42	0.14	0.02	1.06	0.82	1.38	0.66	0.50	1.39	1.04	1.86	0.02	0.67	1.24	0.74	2.06	0.42	5.7E-05	1.14	0.65	2.02	0.66	0.83	1.08	0.85	1.36	0.55	0.39
Fasting insulin	11	1.69	1.16	2.46	0.01	0.24	1.61	1.02	2.55	0.04	0.65	1.65	0.89	3.07	0.11	0.11	2.91	1.15	7.37	0.02	0.25	0.96	0.35	2.60	0.94	0.92	2.26	1.46	3.50	2.9E-04	0.25
Glucose post-2h	6	0.77	0.54	1.09	0.14	0.01	1.27	0.83	1.94	0.27	0.16	0.91	0.58	1.44	0.70	0.64	0.82	0.38	1.77	0.62	0.05	1.98	0.87	4.51	0.11	0.54	1.07	0.75	1.52	0.72	0.31
