# Supplementary material for: Obesity, metabolic factors and risk of different histological types of lung cancer: A Mendelian randomization study
Source: PLoS One. 2017 Jun 8;12(6):e0177875. doi: 10.1371/journal.pone.0177875 (PMC5464539; doi:10.1371/journal.pone.0177875)
Supplement: S3 Table — Int: Intercept. HDL: High-density lipoprotein, LDL: Low-density lipoprotein. Chol: Cholesterol. OR: Odds ratio. Est: Estimate. LCI: Lower confidence interval. UCI: Upper confidence interval. P: P value. (PDF) [file pone.0177875.s024.pdf]

S3 Table - Overall pleiotropic effect assessment of causal estimates of potential risk factors on lung cancer phenotypes provided by MR-Egger test. Int: Intercept. HDL: High-density lipoprotein, LDL: Low-density lipoprotein. Chol: Cholesterol. OR: Odds ratio. Est: Estimate. LCI: Lower confidence interval. UCI: Upper confidence interval. P: P value.

			Lung Overall				Adenocarcinoma				Squamous cell				Small cell				Overall never smokers				Overall ever smokers			
**Risk factor**	**N SNPs**	**MR-Egger parameter**	**Est**	**LCI**	**UCI**	**P**	**Est**	**LCI**	**UCI**	**P**	**Est**	**LCI**	**UCI**	**P**	**Est**	**LCI**	**UCI**	**P**	**Est**	**LCI**	**UCI**	**P**	**Est**	**LCI**	**UCI**	**P**
Body mass index	72	Int	0.01	0.00	0.02	0.02	0.01	-6.E-04	0.02	0.06	0.01	-0.01	0.02	0.33	0.01	-0.01	0.03	0.21	0.00	-0.02	0.03	0.74	0.01	0.00	0.02	4.E-03
		OR	0.77	0.51	1.15	0.20	0.64	0.40	1.02	0.06	0.99	0.55	1.78	0.98	0.99	0.42	2.35	0.98	0.73	0.26	1.99	0.53	0.67	0.43	1.06	0.08
Waist-hip ratio	31	Int	0.00	-0.02	0.02	0.88	0.01	-0.02	0.04	0.55	-0.03	-0.06	0.00	0.06	0.01	-0.04	0.06	0.75	-0.01	-0.07	0.05	0.80	0.01	-0.02	0.03	0.51
		OR	1.13	0.33	3.90	0.84	0.63	0.13	2.95	0.54	5.77	0.83	40.13	0.07	0.80	0.05	12.11	0.87	1.11	0.06	20.47	0.94	0.66	0.16	2.77	0.56
HDL	62	Int	0.00	-0.01	0.00	0.63	0.00	-0.01	0.01	0.98	0.00	-0.01	0.00	0.41	0.00	-0.02	0.01	0.76	0.00	-0.02	0.02	0.92	0.00	-0.01	0.01	0.68
		OR	1.03	0.91	1.16	0.68	1.01	0.88	1.16	0.89	1.07	0.88	1.30	0.51	1.09	0.80	1.48	0.58	1.01	0.70	1.46	0.97	1.05	0.90	1.22	0.53
HDL rare variants	8	Int	0.02	-0.09	0.13	0.70	-0.02	-0.17	0.13	0.74	-0.08	-0.26	0.09	0.30	0.16	-0.12	0.44	0.21	-0.02	-0.36	0.32	0.90	0.06	-0.08	0.20	0.31
		OR	1.01	0.65	1.56	0.97	1.11	0.65	1.89	0.64	1.88	0.81	4.38	0.12	0.45	0.21	0.93	0.04	0.94	0.52	1.71	0.81	0.78	0.55	1.13	0.15
LDL	48	Int	0.01	0.00	0.01	0.13	0.00	-0.01	0.01	0.79	0.01	0.00	0.02	0.01	0.01	-0.01	0.03	0.31	0.01	-0.01	0.03	0.57	0.01	0.00	0.01	0.19
		OR	0.84	0.74	0.96	0.01	0.92	0.77	1.10	0.33	0.72	0.59	0.89	3.E-03	0.85	0.59	1.24	0.40	0.82	0.55	1.23	0.33	0.85	0.73	0.99	0.04
Total Chol	65	Int	0.01	0.00	0.01	0.07	0.01	0.00	0.02	0.11	0.01	0.00	0.02	0.04	0.00	-0.02	0.01	0.81	0.01	-0.01	0.03	0.21	0.00	0.00	0.01	0.39
		OR	0.85	0.73	0.99	0.04	0.90	0.73	1.09	0.28	0.75	0.60	0.95	0.02	0.96	0.65	1.42	0.84	0.79	0.54	1.14	0.20	0.89	0.75	1.06	0.19
Non-HDL rare varia	6	Int	0.02	-0.06	0.10	0.53	0.03	-0.09	0.14	0.54	0.01	-0.14	0.16	0.85	-0.04	-0.24	0.16	0.62	-0.02	-0.27	0.22	0.81	0.04	-0.06	0.15	0.31
		OR	1.00	0.62	1.61	0.98	0.91	0.54	1.55	0.65	1.16	0.57	2.36	0.55	1.19	0.32	4.49	0.73	1.41	0.52	3.83	0.39	0.91	0.44	1.89	0.74
Triglycerides	38	Int	0.00	0.00	0.01	0.27	0.00	-0.01	0.01	0.94	0.01	0.00	0.02	0.12	0.03	0.01	0.04	0.01	0.00	-0.02	0.02	0.81	0.01	0.00	0.02	0.10
		OR	0.92	0.78	1.10	0.35	1.02	0.84	1.23	0.83	0.87	0.65	1.18	0.37	0.64	0.43	0.96	0.03	0.95	0.61	1.49	0.83	0.84	0.68	1.06	0.13
Triglycerides rare va	7	Int	-0.20	-0.36	-0.03	0.03	-0.05	-0.28	0.17	0.58	-0.26	-0.53	0.00	0.05	-0.23	-0.65	0.20	0.23	-0.10	-0.60	0.41	0.64	-0.29	-0.50	-0.07	0.02
		OR	2.70	1.19	6.13	0.03	1.17	0.28	4.91	0.79	3.13	0.65	14.96	0.12	3.75	0.34	41.47	0.22	2.15	0.09	53.66	0.57	4.18	0.62	28.35	0.11
Fasting glucose	24	Int	-0.01	-0.02	0.01	0.30	-0.01	-0.02	0.01	0.30	0.00	-0.02	0.02	0.92	-0.02	-0.04	0.01	0.25	-0.01	-0.04	0.02	0.57	-0.01	-0.02	0.01	0.34
		OR	1.24	0.86	1.80	0.24	1.22	0.83	1.78	0.29	1.36	0.89	2.07	0.15	1.67	0.54	5.20	0.36	1.36	0.66	2.80	0.39	1.29	0.89	1.85	0.17
Fasting insulin	11	Int	-0.02	-0.07	0.03	0.46	0.00	-0.07	0.07	0.95	-0.04	-0.11	0.04	0.32	0.04	-0.08	0.17	0.44	-0.03	-0.18	0.12	0.67	0.00	-0.06	0.06	0.96
		OR	5.32	0.31	91.97	0.22	2.34	0.10	56.51	0.56	18.11	0.13	2.E+03	0.22	0.37	0.00	4.E+02	0.76	4.98	0.03	863.46	0.50	2.33	0.06	92.92	0.62
Glucose post-2h	6	Int	-0.07	-0.14	0.01	0.08	-0.04	-0.15	0.06	0.32	-0.04	-0.17	0.08	0.40	-0.09	-0.28	0.11	0.28	-0.01	-0.24	0.22	0.90	-0.04	-0.14	0.06	0.35
		OR	4.95	0.26	92.75	0.20	3.59	0.11	117.90	0.37	2.50	0.29	21.80	0.31	6.60	0.01	9.E+03	0.51	2.12	0.01	637.70	0.73	2.80	0.21	38.14	0.34
